# Diabetes as a risk factor for pneumococcal disease and severe related outcomes and efficacy/effectiveness of vaccination in diabetic population. Results from meta-analysis of observational studies

**DOI:** 10.1007/s00592-024-02282-5

**Published:** 2024-04-29

**Authors:** Giovanni Antonio Silverii, Giovanni Gabutti, Silvio Tafuri, Francesca Sarti, Anna Pratesi, Alessandra Clerico, Riccardo Fornengo, Carla Greco, Concetta Irace, Valeria Sordi, Gian Pio Sorice, Massimiliano Cavallo, Maria Chantal Ponziani, Edoardo Mannucci, Ilaria Dicembrini, the SID-AMD-SItI Working Group on Diabetes, Vaccines

**Affiliations:** 1 Department of Experimental and Clinical Biomedical Sciences “Mario Serio”, University of Florence, Viale Morgagni 50, 50139 Florence, Italy; 2 Coordinator Working Group “Vaccines and Immunization Policies”, Italian Scientific Society of Hygiene, Preventive Medicine and Public Health (SItI), Terni, Italy; 3 Interdisciplinary Department of Medicine, Aldo Moro, University of Bari, Bari, Italy; 4 Diabetes Unit, Azienda Sanitaria Città di Torino, Turin, Italy; 5 Diabetes Unit, ASL TO4, Chivasso (Turin), Italy; 6 Biomedical and Metabolic Department, Modena and Reggio Emilia University, Modena, Italy; 7 Health Sciences Department, Catanzaro “Magna Graecia” University, Catanzaro, Italy; 8 Diabetes Research Institute, IRCCS San Raffaele Hospital, Milan, Italy; 9 Endocrinology Unit, Bari University Hospital, Bari, Italy; 10 Internal Medicine Unit, Terni “Santa Maria” Hospital, Terni, Italy; 11 Diabetes Unit, Azienda Sanitaria Novara, Novara, Italy

**Keywords:** Diabetes mellitus, Pneumococcal disease, Pneumococcal vaccination

## Abstract

**Aims:**

To collect all available evidence on the effect of diabetes mellitus (DM) as a risk factor for pneumococcal disease incidence and related complications, and on the efficacy/effectiveness of vaccines in patients with DM.

**Methods:**

Two distinct systematic searches on MEDLINE, Cochrane, ClinicalTrials.gov and EMBASE databases were performed, one for each meta-analysis, collecting all observational (cohort and case–control) studies and randomized clinical trials performed on humans up to June 1st, 2023.

**Results:**

We retrieved 36 observational studies comparing risk for pneumococcal disease and related complications in people with or without DM, and 11 studies (1 randomized clinical trial and 10 observational studies) assessing conjugated and polysaccaridic vaccines efficacy/effectiveness on preventing such outcomes. People with DM were at higher risk for Invasive Pneumococcal Disease (unadjusted OR 2.42 [2.00; 2.92]); Case-Fatality Rate (unadjusted OR 1.61 [1.25; 2.07], Pneumococcal pneumonia (unadjusted OR 2.98 [2.76; 3.22), and Intensive care unit admission for pneumococcal disease (unadjusted OR 2.09 [1.20; 3.66]). In diabetic individuals vaccinated with conjugated vaccine, incidence of pneumonia specific for vaccine type in a clinical trial (OR 0.237 [0.008; 0.704]), and hospitalization for overall pneumonia during the year following the polysaccharide vaccination in observational studies (unadjusted OR 0.63 [0.45–0.89]) were significantly lower in comparison with unvaccinated DM subjects, with no significant differences for other outcomes.

**Conclusions:**

People with diabetes mellitus are at higher risk for less favourable course of pneumococcal disease and should be therefore targeted in vaccination campaigns; more evidence needs to be collected on vaccination outcomes in people with diabetes.

**Supplementary Information:**

The online version contains supplementary material available at 10.1007/s00592-024-02282-5.

## Introduction

Pneumococcal disease is a leading cause of hospitalization in the elderly and in patients with chronic comorbidities [1]. Individuals with diabetes (DM) are at increased risk for bacteremic forms of pneumococcal infection with mortality rates as high as 50% [2]. Nowadays, two types of vaccines are available for protection against pneumococcal infections in adults: polysaccharide (23-valent: PPSV23) and conjugate (13-valent: PCV13; 15-valent: PCV15; 20-valent: PCV20) vaccines, PPSV23 following its approval on 1983 through the demonstration of its efficacy on reducing bacteremic pneumonia [3], confirmed its effectiveness in adults against Invasive Pneumococcal Disease [4]. A post-approval clinical trial demonstrated the efficacy of PCV13 vaccination against pneumonia and pneumococcal disease caused by vaccine serotypes in adults aged 65 years and older [5]. PCV15 and PCV20 have been recently licensed by the Food and Drug Administration [6] and the European Medicines Agency [7, 8], and the administration of either PCV20 alone or PCV15 in series with PPSV23 is recommended from 2021 for all adults aged ≥ 65 years, and for adults aged < 65 years with concomitant medical conditions or other risk factors who have not previously received a conjugate vaccine [9]. According to the European Centre for Disease Prevention and Control, the vaccine type recommended in the routine immunization schedules in adults is different across different countries, with PPSV23 and PCV13 currently being the most frequently used, both with single or sequential administration, with conjugate vaccine always preceding polysaccharide vaccine [10]. As for influenza and other preventable diseases, data from observational studies showed coverage rates for pneumococcal vaccination lower than recommended among adults with DM [11, 12].

Clinical recommendations should be based on the systematic analysis of available evidence from properly designed clinical studies; the aim of this manuscript is to define the benefits of pneumococcal vaccination in adults with diabetes. For this purpose, we collected all available evidence (observational studies and/or randomized clinical trials) on the effect of DM as a risk factor for the most severe complications of pneumococcal disease as incidence of Invasive Pneumococcal disease (IPD), case-fatality rate (CFR), intensive care unit (ICU) admission, and on the efficacy/effectiveness of specific vaccines in reducing hospitalization, IPD and mortality in patients with DM.

## Methods

The meta-analyses followed the criteria of Preferred Reporting Items for Systematic Reviews and Meta Analyses (PRISMA) guidelines (Table 1S). Review Protocols were submitted for registration to the PROSPERO website (CRD42023407712 and CRD42023424877 registration numbers, respectively) [13].

### Search strategy

Two distinct systematic searches on MEDLINE, Cochrane, ClinicalTrials.gov and EMBASE databases were performed, one for each meta-analysis, collecting all observational (cohort and case–control) studies and randomized clinical trials performed on humans up to June 1st, 2023. Search terms were reviewed by all collaborators; the full search strings are reported in Table 2S and 3S of supplementary materials. Further studies were manually searched in references from retrieved papers.

### Selection criteria

To be eligible, an item had to be an original report in English of a study enrolling adults with type 1 and/or type 2 DM, assessing selected outcomes.

1. Meta-analysis on Diabetes as a risk factor for pneumococcal disease and related severe complications
  Observational studies of any duration or size were included, provided that they reported data about specific main and additional outcomes, comparing adults with pneumococcal infection affected by versus not affected by Diabetes.
2. Meta-analysis on Pneumococcal vaccine efficacy/effectiveness in Diabetes
  Studies (either observational studies or randomized trials) were included if data about specific main and additional outcomes were available, comparing pneumococcal-vaccinated and non-vaccinated diabetic individuals.

### Endpoints

1. Meta-analysis on Diabetes as a risk factor for complications of pneumococcal disease
  Differences between diabetic and not diabetic adults in incidence of Invasive pneumococcal disease (IPD), case-fatality rate (CFR) and intensive care unit (ICU) admission were the main endpoints, whereas secondary outcomes included differences in incidence of pneumococcal pneumonia, pneumococcal disease, pneumococcal meningitis, pneumococcal septicemia, pneumococcal bacteremia, incidence and length of hospitalization for pneumococcal disease.
2. Meta-analysis on Pneumococcal vaccine effectiveness in Diabetes
  Differences between vaccinated and not vaccinated diabetic adults in hospitalization for pneumonia and for vaccine-type pneumonia, incidence of IPD and vaccine-type IPD were selected as main endpoints, whereas differences between vaccinated and not vaccinated subjects with diabetes in overall hospitalizations and mortality for any cause and for IPD as were selected secondary endpoints.

### Data collection

Titles and abstracts were screened independently by the authors, and potentially relevant articles retrieved in full text. For all published trials, results reported in published papers and supplements were used as the primary source of information. When the required information on protocol or outcomes was not available in the main or secondary publications, an attempt at retrieval was performed consulting the clinicaltrials.gov website. The identification of relevant abstracts and the selection of studies were performed independently by all the authors. Data extraction and conflicts resolution were performed by two investigators (I.D. and G.A.S.). The Cochrane Risk of Bias tool was used to assess risk of bias in randomized controlled trials (RCTs), and the Newcastle–Ottawa Scale was used to assess the risk of bias in observational studies.

### Statistical analyses

Odds ratios and 95% confidence intervals (95% CIs) were either calculated or extracted directly from the publications. Unadjusted or adjusted odds ratio were meta-analyzed separately. Pre-planned separate analyses were performed for randomized trials, whenever possible.

If data from more than one study on a given outcome were available, a meta-analysis using a random-effects model as the primary analysis was performed. Heterogeneity was assessed by using *I*^2^ statistics. Funnel plots were examined to estimate possible publication/disclosure bias, and Egger test was performed to exclude significant publication bias. Sensitivity analyses were performed, whenever possible, if significant heterogeneity was detected, including leave-one out analysis, or subgroup analysis for different time (before/after 2011, year of PCV13 vaccine introduction) or country of observation.

All analyses were performed using Review Manager Web (RevMan Web version 5.3.5) [14] and Comprehensive Meta-analysis [15] software.

Sensitivity analyses were performed, whenever possible, if significant heterogeneity was detected, including leave-one out analysis, or subgroup analysis for different time or country of observation.

## Results

- Diabetes as a risk factor for complications of pneumococcal disease

Study characteristics: Figure 1S, A of Supplementary materials reports the summary flow chart of the meta-analysis. Of the 19,701 items, after removing duplicates, 147 were selected for retrieval of full text. Of those, 111 records were excluded because inclusion criteria were not satisfied (Table 4S). Only 36 studies fulfilled the inclusion criteria. Included studies enrolled 7,740,461 and 83,474,510 patients with and without DM, respectively. Main characteristics of the studies and confounding factors used for statistical adjustment in each of included studies are reported in Table 1. Risk of bias is reported in Table 5S. Two of the included studies [16, 17] reported only subgroups data for different age ranges, therefore we analyzed those age ranges as separate studies.

**Fig. 1:**
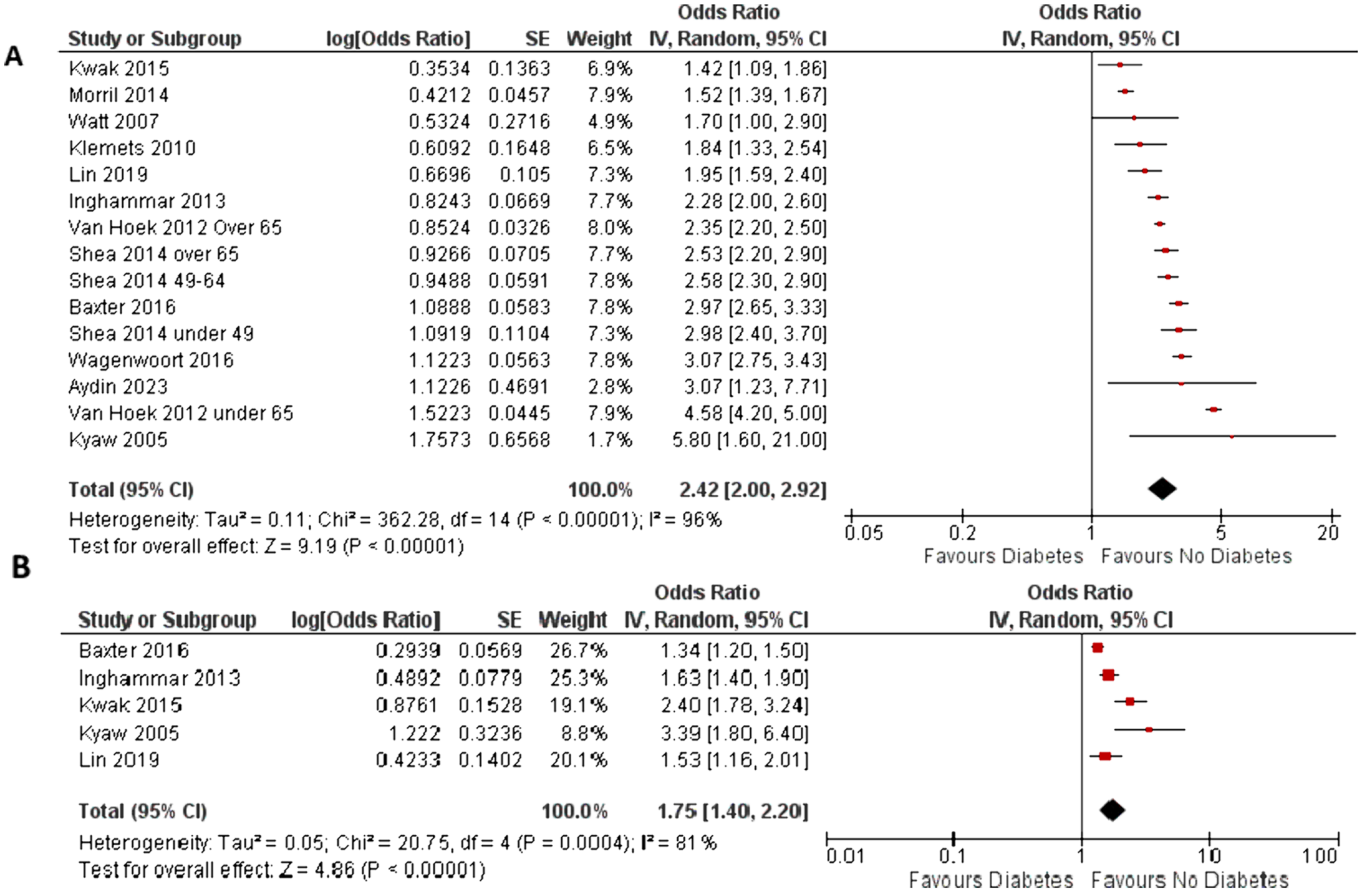
Difference in risk for invasive pneumococcal disease (IPD) between people with or without diabetes mellitus. (IV = inverse variance, CI = confidence interval, SE = standard error); Panel A, Unadjusted OR; Panel B, Adjusted OR)

**Fig. 2:**
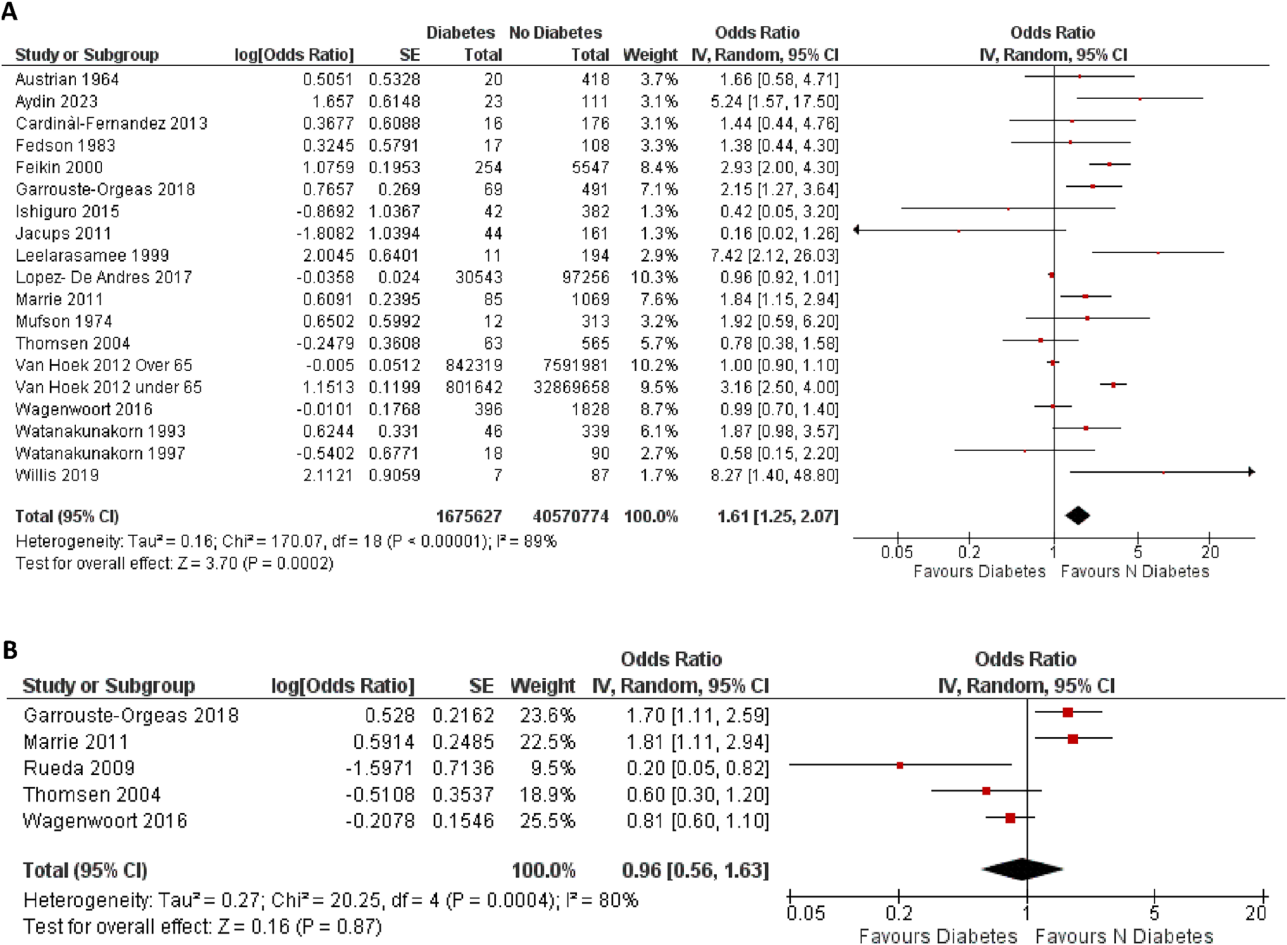
Difference in risk for case-fatality ratio (CFR) between people with or without diabetes mellitus. (IV = inverse variance, CI = confidence interval, SE = standard error); Panel A, Unadjusted OR; Panel B, Adjusted OR)

**Fig. 3:**
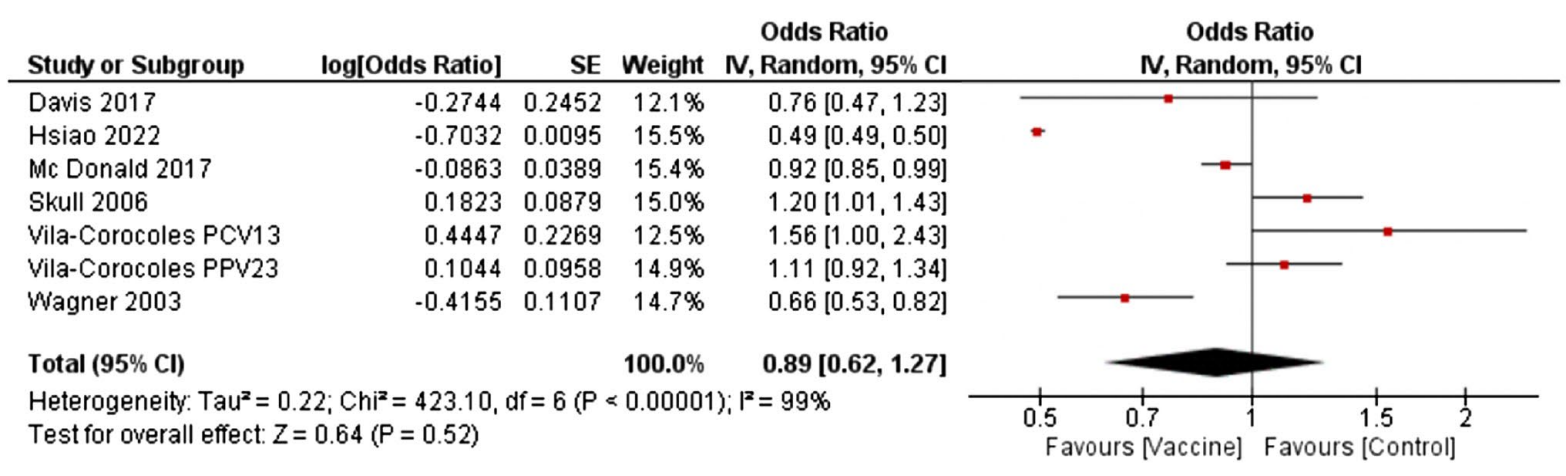
Differences in pneumonia hospitalizations between vaccinated or unvaccinated patients with diabetes (forest plot; IV = inverse variance random = random effects CI = confidence interval)

*Incidence of Invasive Pneumococcal Disease (IPD)*. Twelve of the included studies [2, 16, 17, 19, 20, 29, 32, 34, 36, 39, 43, 44] reported data on this outcome. Funnel plot test (Fig. 2S) ruled out publication bias. DM diagnosis was associated with a significant higher risk of IPD in respect to adults without DM (unadjusted OR 2.42 [2.00; 2.92]; *p* < 0.00001, Fig. 1A), with high heterogeneity. This association was confirmed when available adjusted odds ratios were analyzed (adjusted odds ratio; OR 1.75 [1.40; 2.20]; *p* < 0.00001). Leave-one out analysis was performed, excluding significant influence of single studies (Table 6S). In subgroup analyses, the effect of DM on risk of IPD was significantly greater in studies performed after 2011 when compared to less recent investigations (Fig. 3S); however, the results of studies published after 2011, even when analyzed separately, still showed a relevant heterogeneity. No significant difference in results was observed between cohort and case–control studies (Fig. 4S) and between studies performed in different countries (Fig. 5S). From three studies with available data [16, 17, 43], meta-analysis confirmed a significant association between IPD incidence and subjects aged 65 or older (OR 2.83 [2.10; 3.82]; *p* < 0.00001, Fig. 6S). Meta-regression analyses were also performed in order to explore possible determinants of the effects of DM on incidence of IPD; an inverse not significant association was found with study duration (*r* = − 0.0002 [95%CI − 0.0003; − 0.0000] *p* = 0.052; Fig. 7S), whereas an inverse significant association was found with the proportion of enrolled subjects aged 65 or older (*r* = − 0.004 [95%CI − 0.004; − 0.003] *p* < 0.0001; Fig. 8S). On the other hand, a positive significant correlation was found with the starting observation year (*r* = 0.009 [95%CI 0.002; 0.016] *p* = 0.018; Fig. 9S).

*Case-Fatality Rate (CFR):* Nineteen studies [17–19, 21, 23, 24, 27, 30, 31, 35, 37, 38, 40, 43, 45–48] reported unadjusted odds ratios for this endpoint; the funnel plot did not suggest relevant publication bias (Fig. 10S). DM diagnosis was associated with a significantly higher CFR (unadjusted OR 1.61[1.25; 2.07]; *p* < 0.0002, Fig. 2 A), but the association was no longer confirmed when available adjusted odds ratios were analyzed (Fig. 2B). A leave-one out analysis was performed, ruling out significant influence of single studies (Table 7S). Subgroup analyses showed no difference in risk between studies performed in different countries (Fig. 11S) and between studies performed after 2004, between 1980 and 2004 and before 1980 (Fig. 12S). Meta-regression analyses suggested an inverse association between increase in risk with DM and the proportion of male individuals enrolled (*r* = − 0.04 [95%CI − 0.07; − 0.01] *p* = 0.003; Fig. 13S).

*ICU admission:* Only 2 studies [19, 30] reported unadjusted odds ratio for this endpoint. DM diagnosis was associated with a significant higher ICU admission in respect to individuals without DM (unadjusted odds ratio; OR 2.09 [1.20; 3.66]; *p* = 0.010, Table 8S).

*Incidence of pneumococcal pneumonia:* Five of the included studies reported unadjusted [25, 31, 39, 42] or adjusted [25, 31, 41, 42] odds ratio for this secondary endpoint. DM diagnosis was associated with a significantly higher incidence of pneumococcal pneumonia in respect to individuals without DM (unadjusted OR 2.98 [2.76; 3.22]; *p* < 0.00001, Fig. 14S panel A). The association remained significant when available adjusted odds ratios were analyzed (adjusted odds ratio; adj OR 1.73 [1.46; 2.04]; *p* < 0.00001, Fig. 14S panel B).

*Incidence of overall pneumococcal disease:* Unadjusted odd ratios were retrieved in three studies [26, 41, 49]. DM was not associated with a significant increase in risk for overall pneumococcal disease in the general population (unadjusted OR 2.02 [0.64; 5.49]; *p* = 0.23, Table 8S), whereas a significant association was found in patients aged 65 years or older (unadjusted OR 1.56 [1.44; 1.71]; *p* < 0.00001, Table 8S).

*Incidence of pneumococcal bacteremia, septicemia and meningitis:* Two studies [22, 33, 39, 41] reported data for the aggregate of pneumococcal bacteremia, septicemia and meningitis. DM was associated with a significant increase of incidence for this composite endpoint in adjusted, but not in unadjusted analysis (unadjusted OR 1.16 [0.56; 2.41]; *p* = 0.70, Fig. 18S; adjusted OR 1.48 [1.08; 2.04]; *p* = 0.01, Table 8S).

*Incidence and length of stay of hospitalization for pneumococcal disease:* None of the included studies reported data on these outcomes.

- Pneumococcal vaccine efficacy/effectiveness in diabetes.

*Study characteristics:* Fig. 1S, B of Supplementary materials reports the summary flowchart of the meta-analysis. Of the 19,701 items, after removing duplicates, 19,611 were selected for retrieval of full text. Of those, 19,518 records were excluded after reading abstract, because inclusion criteria were not satisfied, whereas 82 were excluded after full text examination (reason for exclusion are reported in Table 9S). Only 11 studies fulfilled the inclusion criteria overall enrolling 600,074 individuals: 10 observational studies (of which eight performed with PPSV23, one with PCV13, and one including both vaccines) and one clinical trial, performed with PCV13. The risk of bias table is reported in Table 10S; the main characteristics of included studies are reported in Table 2; confounding factors used for statistical adjustment in each of included studies are reported in Table 10S.

**Table 1:** Characteristics of the studies included in the meta-analysis on the effect of diabetes mellitus on pneumococcal disease

Study name	Dur	Years obs	Cou	Pat	Type	Age	> 65	M	DM	Not DM
Austrian [18]	520	1952–1962	USA	Hosp	CH	–	–	–	20	418
Aydin [19]	520	2012–2021	TWN	H/P	CH	–	40	63	23	111
Baxter [20]	364	2008–2014	USA	Pop	CH	–	20		1585715	13516332
Cardinal [21]	156	2008–2010	URY	Hosp	CH	55	–	55	16	176
Di Yacovo [22]	444	2002–2010	ESP	Hosp	CH	71	–	68	516	1891
Fedson [23]	479	1970–1980	USA	Hosp	CH	–	–	–	17	108
Feikin [24]	156	1995–1997	USA	Hosp	CH	–	40	54	254	5547
Flory [25]	78	2002–2004	USA	Pop	CH	–	51	46	253,266	2,685,074
Fukuda [26]	52	2015–2016	JPN	Pop	CH	–	49	–	35,355	696,880
Garrouste [27]	1040	1997–2016	FRA	Hosp	CH	59	–	62	69	491
Gil-Prieto [28]	52	2011–2011	ESP	H/P	CH	71	70	60	209,540	–
Inghammar [29]	988	1990–2008	SWE	Pop	CC	67	–	49	1667	42,771
Ishiguro [30]	728	2002–2015	JPN	Hosp	CH	69	67	69	42	382
Jacups [31]	1144	1987–2008	AUS	Pop	CH	–	–	61	4739	82,266
Klemets [32]	416	1995–2002	FIN	Pop	CC	38	0	68	293	13,774
Kornum [33]	416	1997–2004	DNK	Hosp	CH	–	69	53	3023	26,877
Kwak [34]	52	2011–2011	SKR	Pop	CH	–	0	–	104,769	847,526
Kyaw [2]	104	1999–2000	USA	Pop	CH	–	–	–	3942	56,089
Leelarasamee [35]	384	1992–1998	THA	Hosp	CH	–	28	63	11	194
Lin [36]	728	2000–2013	TWN	Pop	CC	–	23	63	495	4005
Lipsky [26]	290	1977–1982	USA	Pop	CC	–		100	40	153
Lòpez [37]	520	2004–2013	ESP	Hosp	CH	–		61	30,543	97,256
Marrie [38]	260	2000–2004	CAN	Hosp	CH	–		55	85	1069
Morrill [39]	520	2002–2011	USA	Hosp	CH	68		98	2344	12,167
Mufson [40]	208	1967–1970	USA	Hosp	CH	–		76	12	313
Rueda [65]	378	2000–2007	USA	Hosp	CC	64		98	53	180
Seminog [41]	260	2007–2011	UK	Pop	CH			–	2,244,109	8,976,436
Shea [16]	260	2006–2010	USA	Pop	CH	–		–	7,988,291	78,795,527
Thomsen [33]	520	1992–2001	DNK	Hosp	CH	68		48	63	565
Van Hoek [17]	364	2002–2009	UK	Pop	CH	–	–	–	1,643,961	40,461,639
Vila-Corcoles [42]	156	2008–2011	ESP	Pop	CH	72	–	45	5905	21,299
Wagenvoort [43]	208	2008–2012	NLD	Pop	CH	–	19	–	11,730	162,544
Watt [44]	113	1999–2002	USA	Pop	CC	52	–	48	115	356
Watanakunakorn [45]	520	1980–1989	USA	Hosp	CH	48	43	52	46	339
Watanakunakorn [46]	226	1992–1996	USA	Hosp	CH	–	61	41	18	90
Willis [47]	104	2008–2009	JAM	Hosp	CH	–	–	–	7	87

Numbers in the study of Shea et al. are expressed in patient-years

BMI = body mass index, PORT score = pneumonia severity index, COPD = chronic obstructive pulmonary disease, MPR = medication possession ratio. CC = Case–control; CH = Cohort; Dur = duration (in weeks) OBS = duration.

**Table 2:** Characteristics of the studies included in the meta-analysis on the effect of pneumococcal vaccination in people with DM

Study	Dur	Years	Cou	Type	Age	M	> 65	Vaccine	Vaccinated	
									Yes	No
Benin [50, 51]	104	1996–1997	USA	CC	59	55	100	PPV23	155	287
Butler [50, 52]	728	1978–1992	USA	CH	–	–	–	PPV23-PPV14	330	904
Davis [53, 54]	260	2008–2013	AUS	CH	65	54	–	PPV23	624	841
Fisman [55]	221	1999–2003	USA	CH	73	53	–	PPV23	1877	3402
Huiits [5]	260	2008–2013	NL	RCT	71	59	100	PCV13	2928	2958
Hsiao [22, 53]	156	2015–2018	USA	CH		44	100	PCV13	20,062	5117
Kuo [56]	156	2007–2009	TW	CH	81	52	100	PPV23	33,395	33,395
Mc Donald [51]	780	1997–2011	UK	CH	72	51	100	PPV23	27,584	189,776
Skull [57]	104	2000–2002	AUS	CH	77	54	100	PPV23	872	374
Vila-Corocoles [58]	104	2015–2016	ESP	CH	–	46		PPV23-PCV13	25,278	442,047
Wagner [59]	116	1997–1998	AUT	CC	82		100	PPV23	514	563

BMI = body mass index, PORT score = pneumonia severity index, COPD = chronic obstructive pulmonary disease, MPR = medication possession ratio. Case–control = CC; CH = Cohort. Numbers from Mc Donald 2017 are expressed as patient-years. Dur = duration

*Hospitalization for vaccine-type pneumonia:* In the only randomized trial available [5], PCV13 vaccine was associated with a significantly lower hospital admission for vaccine-type pneumonia in diabetic individuals aged over 65 years (OR 0.237 [0.008; 0.704]; *p* = 0.002), as reported in Fig. 15S. No data from observational studies were available for this endpoint.

*Hospitalization for overall pneumonia:* Six observational studies [51, 53, 54, 57–59] reported unadjusted odds ratio for this endpoint, reporting no association between pneumococcal vaccination and risk of hospitalization for overall pneumonia in diabetic individuals (OR 0.89 [0.62; 1.27]; *p* = 0.52, Fig. 3), with high heterogeneity (*I*^2^ = 99%). A funnel plot did not allow to rule out publication bias, with studies providing more positive results for vaccine having a greater chance of being published (Fig. 16S). No difference was found between studies performed in USA and Australia in comparison with those performed in Europe (*p* = 0.54; Fig. 17S) and between PCV13 and PPV23 vaccines (*p* = 0.89; Fig. 18S).

In three studies with available data 1 year or less following vaccination [51, 53, 59], all performed with PPV23 vaccines, hospitalization for overall pneumonia resulted significantly lower in vaccinated versus not vaccinated adults with diabetes (OR 0.63 [0.45–0.89;*p* = 0.008; Fig. 19S).

*Incidence of Invasive Pneumococcal Disease (IPD) and vaccine-type IPD:* Only one observational study [50] reported data on this endpoint, showing an association of PPV23 vaccination with a lower risk of vaccine-type IPD (OR 0.81 [0.25–2.57]). In three studies with available data [50, 52, 56], PPV pneumococcal vaccination was not associated with incidence of overall IPD (including cases of IPD from serotypes different from those targeted by vaccination; OR 0.55 [0.24–1.26]; *p* = 0.15; Fig. 20S]. In the only study of those reporting odds ratios adjusted for confounders [56], the incidence of IPD was significantly reduced in vaccinated versus unvaccinated adults with DM (adj OR 0.85 [0.77–0.93]).

*Overall hospitalizations:* This endpoint was reported only in one study [56], showing a significant association of pneumococcal vaccination with a reduction of overall hospitalization rates, in both unadjusted (OR 0.94 [0.91–0.98]) and adjusted (OR 0.96 [0.92–0.99]) analysis.

*Overall mortality.* Two of selected studies reported adjusted odds ratio for this endpoint [55, 58], with no significant association between vaccination and overall mortality (OR 0.98 [0.93–1.04; *p* = 0.55; Fig. 21S).

*Mortality for IPD.* None of the included studies reported data on this outcome.

## Discussion

The present meta-analysis shows that DM is associated with an increased risk of pneumococcal disease and related severe outcomes, with a two–threefold greater incidence of IPD in adults with diabetes in comparison with the general population. This risk remains significantly higher also after adjusting for potential confounders. However, results of studies are heterogeneous, prompting further analyses for the assessment of potential moderators. Meta-regression and subgroup analyses suggest that the association may be stronger in the elderly; furthermore, the effect of DM seems to be greater in more recent studies; accordingly, a previously published meta-analysis [60], detected a weaker, although significant, association between diabetes and the risk for IPD and pneumococcal pneumonia. The mechanisms underlying this associations, which are beyond the aim of this paper, may include coinfections with other agents, impairment of immune responses, chronic inflammation associated with hyperglycemia and/or insulin resistance, and other mechanisms [61].

In people with diabetes, a post hoc analysis of the only available randomized clinical trial [5] showed that pneumococcal conjugate vaccine PCV13 was effective in reducing hospitalizations for pneumonia determined by serotypes targeted by the specific vaccine used; on the other hand, our meta-analysis of observational studies failed to demonstrate the effectiveness of the pneumococcal vaccines in reducing IPD, hospitalizations or mortality in this population in observational studies, with no significant difference detected between PPSV23 and PCV13.

Furthermore, our subgroup analysis of available observational studies suggested the efficacy of PPSV23 vaccine against hospitalization for pneumonia in adults with diabetes one year or less after vaccination, confirming thus a likely reduction in the efficacy of this type of vaccine over the time, which had already been observed with PPSV23, especially in the elderly [62]. A previous, systematic review exploring differences in pneumococcal-related outcomes in vaccinated adults with and without diabetes, including a smaller number of studies, also provided conflicting results [63].

The results of observational studies seem to question the effectiveness of pneumococcal vaccines in people with diabetes. On the other hand, the only available randomized trial indicates that vaccination is effective in individuals with diabetes [5]. Although the result in diabetes derives from a post hoc analysis, with the risk of selective publication of positive results, data from a randomized trial have a higher level of evidence than observational studies. In fact, the ability of observational studies in detecting the true effect of a treatment is severely limited by potential residual confounding, mainly prescription bias: vaccinated individuals with DM may have a higher baseline risk for complications than those who were not vaccinated, which adjustments may not fully address; such impairment could possibly interfere with the estimates of effectiveness [58]. A further possible explanation of the reduced efficacy of vaccination may rely on the increased prevalence, in the population enrolled in the included studies, of pneumococcal non-vaccine serotypes [58].

In order to explore the need for promoting pneumococcal vaccination in people with diabetes, a cost-effectiveness analysis is needed. Such analysis should rely on accurate and updated data about incidence and severity of infection due to each pneumococcal serotype; therefore, the serotype determination in IPD should be strongly encouraged. However, the determination of actual infection rates is a relevant organizational challenge. Notably, the rate of serotype determinations has been decreasing from 2019 [64], probably due to the consequences of the need for the healthcare system to focus their resources on the COVID-19 pandemic. In order to assess the efficacy of vaccines, more high-quality data are strongly needed, ideally from randomized clinical trials. Although several clinical trials on pneumococcal vaccination have been performed, only one provided separate data for people with diabetes; conversely, subgroup analyses for all the categories considered at higher risk for pneumococcal complications should be performed in all trials, to confirm that comorbidities do not affect vaccine efficacy.

All available evidence refers to the association of pneumococcal disease outcomes with PPSV23 and PCV13 vaccines. PCV15 and PCV20 have been reported to be more effective than previous vaccines [6], but no specific data were available in people with diabetes. Further limitations should be considered in the interpretation of this meta-analysis: many results showed a high heterogeneity, which could be only partly explained by factors identified as moderators. In fact, specific subgroup data for other variables (i.e., type of diabetes, pharmacologic treatment, glucose control, comorbidities) were unavailable. In many of the subgroup analyses performed, the low number of studies included should be considered as a potential bias regarding the risk evaluation. Moreover, a confounding bias related to previous influenza vaccination is also possible, since one of the most frequent complications of influenza is a pulmonary pneumococcal infection.

In conclusion, the present systematic review and meta-analysis shows that: (1) adults with diabetes showed higher risk of pneumococcal disease and severe related complications versus not diabetic individuals and (2) in people with diabetes, pneumococcal vaccination appears to be effective in preventing vaccine-type pneumonia in clinical trials.

### Supplementary Information

Below is the link to the electronic supplementary material.

Supplementary file1 (PDF 1368 KB)
