# Supplementary material for: Diabetes as a risk factor for pneumococcal disease and severe related outcomes and efficacy/effectiveness of vaccination in diabetic population. Results from meta-analysis of observational studies
Source: Acta Diabetol. 2024 Apr 29;61(8):1029–39. doi: 10.1007/s00592-024-02282-5 (PMC11329702; doi:10.1007/s00592-024-02282-5)
Supplement: Supplementary file 1 — Supplementary file1 (PDF 1368 KB) [file 592_2024_2282_MOESM1_ESM.pdf]

Supplementary Materials

		PRISMA 2020 Checklist
**Section and Topic**	**Item #**	**Checklist item**
**TITLE**		
Title	1	Identify the report as a systematic review.<br>*Title: "Diabetes as a risk factor for Pneumococcal disease and severe related-outcomes and efficacy/effectiveness of vaccination in diabetic population. Results from meta-analysis of observational studies".*
**ABSTRACT**		
Abstract	2	See the PRISMA 2020 for Abstracts checklist.
**INTRODUCTION**		
Rationale	3	Describe the rationale for the review in the context of existing knowledge.<br>Individuals with diabetes (DM) are at increased risk for bacteremic forms of pneumococcal infection with mortality rates as high as 50% and Pneumococcal disease is a leading cause of hospitalization in the elderly and in patients with chronic comorbidities.
Objectives	4	Provide an explicit statement of the objective(s) or question(s) the review addresses.<br>*"In order to better define the need for pneumococcal vaccination in people with diabetes, we collected all available evidence on the effect of DM as a risk factor for pneumococcal-related complications, and on the specific efficacy of vaccines in patients with DM.*
**METHODS**		
Eligibility criteria	5	Specify the inclusion and exclusion criteria for the review and how studies were grouped for the syntheses.<br>To be eligible, an item had to be an original report in English of a study enrolling adults with type 1 and/or type 2 DM, assessing selected outcomes.
Information sources	6	Specify all databases, registers, websites, organisations, reference lists and other sources searched or consulted to identify studies. Specify the date when each source was last searched or consulted.<br>Two distinct systematic searches on MEDLINE, Cochrane, ClinicalTrials.gov and Embase databases were performed, one for each meta-analysis, collecting all observational (cohort and case-control) studies and randomized clinical trials performed on humans up to June 1st, 2023.
Search strategy	7	Present the full search strategies for all databases, registers and websites, including any filters and limits used.<br>Search terms were reviewed by all collaborators; the full search strings are reported in Table 2S and 3S of supplementary materials. Further studies were manually searched in references from retrieved papers
Selection process	8	Specify the methods used to decide whether a study met the inclusion criteria of the review, including how many reviewers screened each record and each report retrieved, whether they worked independently, and if applicable, details of automation tools used in the process.<br>*"Titles and abstracts were screened independently by two authors, and potentially relevant articles were retrieved in full text. For all published trials, results reported in published papers and supplements were used as the primary source of information; when the required information on protocol or outcomes was not available in the main publication secondary publications were used for retrieval of missing information; whenever needed an attempt at retrieval of missing information was performed consulting the clinicaltrials.gov registry".*
Data collection process	9	Specify the methods used to collect data from reports, including how many reviewers collected data from each report, whether they worked independently, any processes for obtaining or confirming data from study investigators, and if applicable, details of automation tools used in the process. *"Titles and abstracts were screened independently*

		by the authors, and potentially relevant articles retrieved in full text. For all published trials, results reported in published papers and supplements were used as the primary source of information. When the required information on protocol or outcomes was not available in the main or secondary publications, an attempt at retrieval was performed consulting the clinicaltrials.gov website. The identification of relevant abstracts and the selection of studies were performed independently by all the authors. Data extraction and conflicts resolution were performed by two investigators (I.D. and G.A.S.). The Cochrane Risk of Bias tool was used to assess risk of bias in Randomized Controlled Trials (RCTs) and the Newcastle-Ottawa Scale was used to assess the risk of bias in observational studies.
Data items	10a	List and define all outcomes for which data were sought. Specify whether all results that were compatible with each outcome domain in each study were sought (e.g. for all measures, time points, analyses), and if not, the methods used to decide which results to collect.<br>*Endpoints:*<br>*1. Metanalysis on Diabetes as a risk factor for complications of pneumococcal disease*<br>Differences between diabetic and not diabetic adults in incidence of Invasive Pneumococcal Disease (IPD), Case-Fatality Rate (CFR), Intensive Care Unit (ICU)-admission were the main endpoints, whereas secondary outcomes included differences in incidence of pneumococcal pneumonia, pneumococcal disease, pneumococcal meningitis, pneumococcal septicaemia, pneumococcal bacteremia, incidence and length of hospitalization for pneumococcal disease.<br>*0. Metanalysis on pneumococcal vaccine efficacy in diabetes*<br>Differences between vaccinated and not vaccinated diabetic adults in hospitalization for pneumonia and for vaccine-type pneumonia, incidence of IPD and vaccine-type IPD were selected as main endpoints whereas differences between vaccinated and not vaccinated subjects with diabetes in overall hospitalizations and mortality for any cause and for IPD as secondary endpoints.
	10b	List and define all other variables for which data were sought (e.g. participant and intervention characteristics, funding sources). Describe any assumptions made about any missing or unclear information. *"See Item 10a"*
Study risk of bias assessment	11	Specify the methods used to assess risk of bias in the included studies, including details of the tool(s) used, how many reviewers assessed each study and whether they worked independently, and if applicable, details of automation tools used in the process. *"The Cochrane Risk of Bias tool was used to assess risk of bias in Randomized Controlled Trials (RCTs) and the Newcastle-Ottawa Scale was used to assess the risk of bias in observational studies"*
Effect measures	12	Specify for each outcome the effect measure(s) (e.g. risk ratio, mean difference) used in the synthesis or presentation of results. *"Odds ratios and 95% confidence intervals (95% CIs) were either calculated or extracted directly from the publications. Unadjusted or adjusted odds ratio are meta-analysed separately, using the DerSimonian-Laird method. Pre-planned separate analyses were performed for randomized trials, whenever possible.".*
Synthesis methods	13a	Describe the processes used to decide which studies were eligible for each synthesis (e.g. tabulating the study intervention characteristics and comparing against the planned groups for each synthesis (item #5)). *See Item 5*
	13b	Describe any methods required to prepare the data for presentation or synthesis, such as handling of missing summary statistics, or data conversions. *See Item 10a*
	13c	Describe any methods used to tabulate or visually display results of individual studies and syntheses. *"If data from more than one study on a given outcome were available, a meta-analysis using a random-effects model as the primary analysis was performed."*
	13d	Describe any methods used to synthesize results and provide a rationale for the choice(s). If meta-analysis was performed, describe the model(s), method(s) to identify the presence and extent of statistical heterogeneity, and software package(s) used. *"Heterogeneity was assessed by using I2 statistics. Funnel plots were examined in order to estimate possible publication/disclosure bias. All analyses were performed using Review Manager.*<br>*"Odds ratios and 95% confidence intervals (95% CIs) were either calculated or extracted directly from the publications. Unadjusted or adjusted odds ratio are meta-analysed*

		separately, using the DerSimonian-Laird method. Pre-planned separate analyses were performed for randomized trials, whenever possible."
	13e	Describe any methods used to explore possible causes of heterogeneity among study results (e.g. subgroup analysis, meta-regression). "If data from more than one study on a given outcome were available, a meta-analysis using a random-effects model as the primary analysis was performed. Heterogeneity was assessed by using I2 statistics. Funnel plots were examined in order to estimate possible publication/disclosure bias. All analyses were performed using Review Manager.
	13f	Describe any sensitivity analyses conducted to assess robustness of the synthesized results. *"To evaluate the influence of each study on the overall effect size, sensitivity analysis was conducted using the one study remove (leave-one-out) approach*"
Reporting bias assessment	14	Describe any methods used to assess risk of bias due to missing results in a synthesis (arising from reporting biases).<br>The Cochrane Risk of Bias tool was used to assess risk of bias in Randomized Controlled Trials (RCTs) and the Newcastle-Ottawa Scale was used to assess the risk of bias in observational studies. PRISMA flow diagram for search and selection processes of the meta-analysis has been applied .
Certainty assessment	15	Describe any methods used to assess certainty (or confidence) in the body of evidence for an outcome.
**RESULTS**		
Study selection	16a	Describe the results of the search and selection process, from the number of records identified in the search to the number of studies included in the review, ideally using a flow diagram. *See Supplementary materials.*
	16b	Cite studies that might appear to meet the inclusion criteria, but which were excluded, and explain why they were excluded.<br>Supplementary materials reported the trial flow summary and reasons of exclusion for unselected studies.
Study characteristics	17	Cite each included study and present its characteristics. "<br>*<u>Metanalysis on Diabetes as a risk factor</u>*<br>*Only 36 studies fulfilled the inclusion criteria. Included studies enrolled 7,740,461 and 83,474,510 patients with and without DM, respectively. Main characteristics of the studies and confounding factors used for statistical adjustment in each of included studies are reported in Table 1. Risk of bias is reported in table 5S. Two of the included studies reported only subgroups data for different age ranges, therefore we analyzed those age ranges as separate studies.*<br><u>Metanalysis on Pneumococcal vaccine efficacy in diabetes.</u><br>Only 11 studies fulfilled the inclusion criteria overall enrolling 600,074 individuals: 10 observational studies (of which eight performed with PPSV23, one with PCV13, and one including both vaccines) and one clinical trial, performed with PCV 13. The risk of bias table is reported in Table 10S; the main characteristics of included studies are reported in Table 2;
Risk of bias in studies	18	Present assessments of risk of bias for each included study. "*The risk of bias is reported in supplementary materials".*
Results of individual studies	19	For all outcomes, present, for each study: (a) summary statistics for each group (where appropriate) and (b) an effect estimate and its precision (e.g. confidence/credible interval), ideally using structured tables or plots.<br>*<u>Metanalysis on Diabetes as a risk factor</u>*<br>*See Results section pages 6-8 of manuscript for each endpoint.*<br>*<u>Metanalysis on Defficacy of Pneumococcal vaccine in diabetes</u>*<br>*See Results section pages 8-10 of manuscript for each endopoint.*
Results of syntheses	20a	For each synthesis, briefly summarise the characteristics and risk of bias among contributing studies. See item 18;

	20b	Present results of all statistical syntheses conducted. If meta-analysis was done, present for each the summary estimate and its precision (e.g. confidence/credible interval) and measures of statistical heterogeneity. *See item 19, and supplementary materials*
	20c	Present results of all investigations of possible causes of heterogeneity among study results. . "*A sensitivity analysis was performed to explore heterogeneity, excluding one trial at a time, which confirmed the results; (see supplementary materials)".*
	20d	Present results of all sensitivity analyses conducted to assess the robustness of the synthesized results. "*A sensitivity analysis was performed to explore heterogeneity, excluding one trial at a time, which confirmed the results; (see supplementary materials)".*
Reporting biases	21	Present assessments of risk of bias due to missing results (arising from reporting biases) for each synthesis assessed. *See item 18*
Certainty of evidence	22	Present assessments of certainty (or confidence) in the body of evidence for each outcome assessed. *See item 18*
**DISCUSSION**		
Discussion	23a	*Provide a general interpretation of the results in the context of other evidence.*<br>The present meta-analysis confirms that DM is associated with an increased risk of complications of pneusmococcal disease, and that vaccination is effective for preventing such complications in people with diabetes.
	23b	Discuss any limitations of the evidence included in the review.<br>… the ability of observational studies in detecting the true effect of a treatment is severely limited by potential residual confounding, mainly prescription bias: vaccinated individuals with DM may have a higher baseline risk for complications than those who were not vaccinated, which adjustments may not fully address; such impairment could possibly interfere with the estimates of effectiveness….. In many of the subgroup analysises performed the low number of studies included should be considered as a potential bias regarding the risk evaluation. Moreover, a confounding bias related to previous influenza vaccination is also possible, since one of the most frequent complications of influenza is a pulmonary pneumococcal infection.
	23c	Discuss any limitations of the review processes used.<br>Further limitations should be considered in the interpretation of this meta-analysis: many results showed a high heterogeneity, which could be only partly explained by factors identified as moderators
	23d	Discuss implications of the results for practice, policy, and future research.<br>the present systematic review and meta-analysis shows that: 1) Pneumococcal disease is associated with more severe complications in diabetic versus not diabetic individuals and 2) penumococcal vaccination is effective in preventing clinically relevant outcomes in adults with DM. The identification of patients with diabetes as the target of vaccination campaigns for pneumococcal appears to be justified by available clinical evidence.
**OTHER INFORMATION**		
Registration and protocol	24a	Provide registration information for the review, including register name and registration number, or state that the review was not registered*. "Review Protocol was submitted for registration to the PROSPERO website (CRD42023407712 and CRD42023424877 registration numbers, respectively)*
	24b	Indicate where the review protocol can be accessed, or state that a protocol was not prepared*. See item 24a*
	24c	Describe and explain any amendments to information provided at registration or in the protocol*. See item 24a*
Support	25	Describe sources of financial or non-financial support for the review, and the role of the funders or sponsors in the review.<br>*"Funding and data transparency This research was performed as a part of the institutional activity of the unit, with no specific funding".*
Competing interests	26	Declare any competing interests of review authors.<br>***Potential Conflicts of interest:*** *GG declares grants from Sanofi Pasteur MSD, GSK Biologicals SA, Pfizer, Sanofi Pasteur, MSD Italy, Emergent BioSolutions, Moderna,*

		Novavax and Seqirus for taking part to advisory boards, expert meetings, for acting as speaker and/or organizer of meetings/congresses and as principal investigator and chief of O.U. in RCTs. All the others authors have no conflict of interest to disclose directly related to this manuscript.
Availability of data, code and other materials	27	Report which of the following are publicly available and where they can be found: template data collection forms; data extracted from included studies; data used for all analyses; analytic code; any other materials used in the review. *"Data availability Statement: The authors confirm that the data supporting the findings of this study are available within the article and its supplementary materials."*

Table 1S: The Prisma Statement 2020 checklist

**EMBASE** Search:_(‘Invasive pneumococcal disease’ OR ‘IPD’ OR ‘pneumococcal septicaemia’ OR ‘lobar pneumonia’ OR ‘pneumococcal meningitis’ OR ‘pneumococcal pneumonia’ OR bacterial pneumonia’ OR ‘pneumococcal bacteriemia’ OR ‘s. pneumoniae’ OR ‘streptococcus pneumoniae’) AND ('diabetes mellitus'/exp OR 'diabetes mellitus') AND ('mortality'/exp OR mortality OR 'morbidity'/exp OR morbidity OR 'disease severity'/exp OR 'disease severity' OR 'disease predisposition'/exp OR 'disease predisposition' OR risk OR 'risk factor' OR ‘incidence’
MEDLINE Search: (‘Invasive pneumococcal disease’ OR ‘IPD’ OR ‘pneumococcal septicaemia’ OR ‘lobar pneumonia’ OR ‘pneumococcal meningitis’ OR ‘pneumococcal pneumonia’ OR bacterial pneumonia’ OR ‘pneumococcal bacteriemia’ OR ‘s. pneumoniae’ OR ‘streptococcus pneumoniae’) AND (diabetes mellitus OR diab*) AND (mortality OR morbidity OR disease severity OR susceptibility OR risk OR risk factor) Filters: Humans Sort by: Most Recent

Table 2S: Information on search string, effect of diabetes mellitus on pneumococcal disease severity

**EMBASE** Search: (‘pneumococcal vaccin*’ OR ‘IPD’ OR ‘pneumococcal vaccin*’ OR ‘pneumococcal immuniz*’ OR ‘pneumococcal vaccination’ OR ‘pneumococcal immunization’’) AND ('diab*'/exp OR 'diabetes mellitus') AND OR 'streptococc*/exp vaccination' OR ' streptococc*/exp 'vaccin*'/exp)
**MEDLINE** Search: (‘pneumococcal vaccin*’ OR ‘IPD’ OR ‘pneumococcal vaccine’ OR ‘immuniz*’ OR ‘pneumococcal vaccination*’ OR ‘pneumococcal immunization*’ OR ‘pneumococcal bacteriemia’ OR ‘strptococcal immunizatione’ OR ‘streptococcus vaccination’) AND (diabetes mellitus OR diab*) Filters: Humans
**CLINICALTRIALS**.GOV : Pneumococcal vaccine AND diabetes mellitus
COCHRANE CENTRAL: Pneumococcal vaccine AND diabetes mellitus

Table 3S: Information on search string, pneumococcal vaccine efficacy in people with diabetes

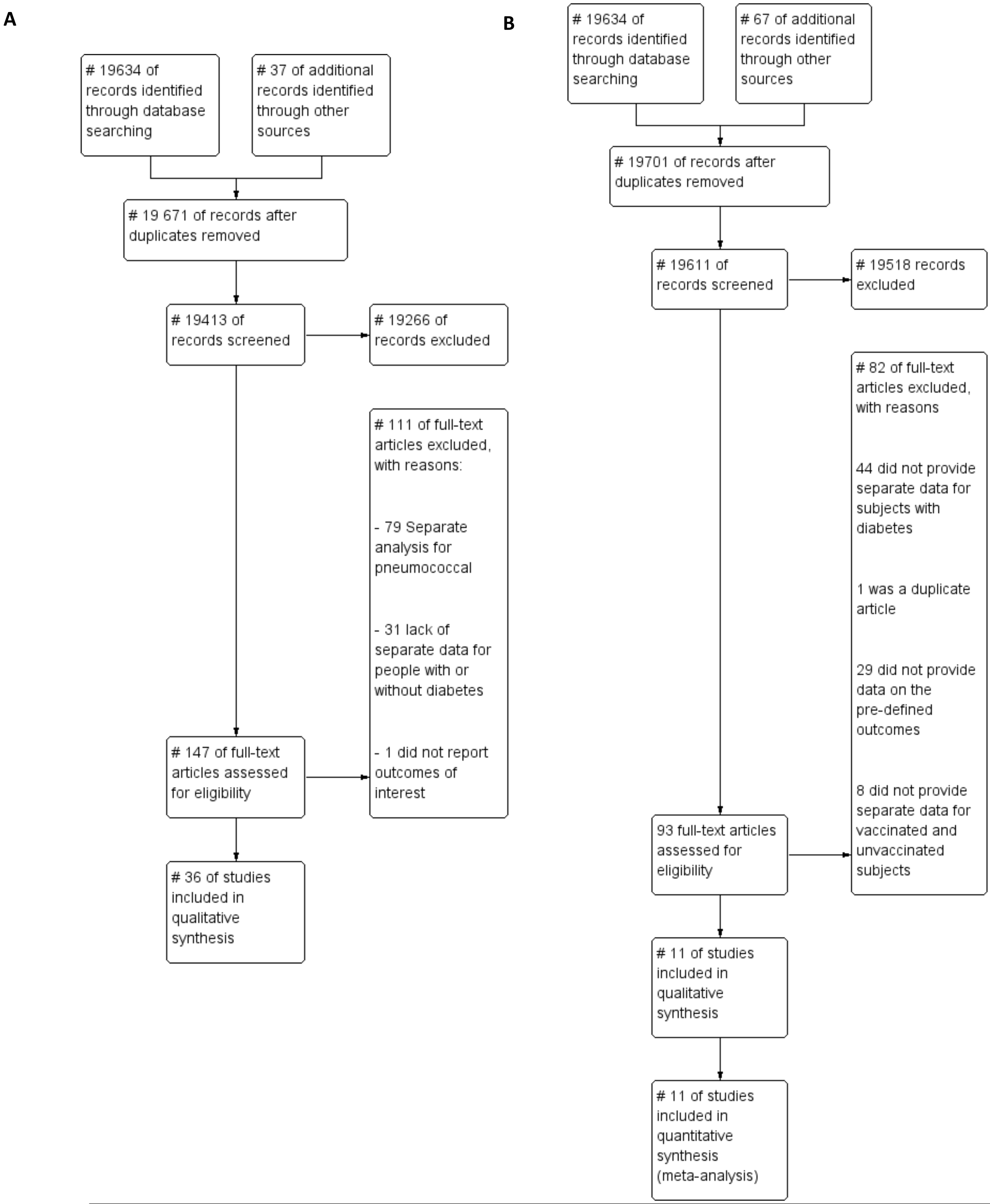

Figure 1S: Flow chart. **A:** effect of diabetes mellitus on pneumococcal disease severity;

B: effectiveness of pneumococcal vaccine in people with diabetes.

Study	Year	Reason for exclusion
Alfageme	2006	No separate data for people with Diabetes
Almusalam	2019	No outcomes
Andrews	2012	No separate data for people with Diabetes
Ansladi	2005	No separate data for people with Diabetes
Appel	2006	No outcomes
Aubert	2010	No outcomes
Bardenheier	2022	No outcomes
Bliss	2008	No separate data for people with Diabetes
Boey	2021	No outcomes
Chandler	2022	No separate data for people with Diabetes
Chen YH	2006	No outcomes
Chevalier-Cottin	2020	No outcomes
Christenson	2001	No separate data for people with Diabetes
Christenson	2004	No separate data for people with Diabetes
Christenson	2008	No separate data for people with Diabetes
Demirci	2021	No outcomes
Djennad A	2019	No separate data for people with Diabetes
Dominguez	2005	No separate data for people with Diabetes
Dorner	2011	No outcomes
Dower	2011	No outcomes
Furumoto	2008	No separate data for people with Diabetes
Gessner	2019	Double
Gil-Prieto R	2016	No vaccination rate
Gilbertson	2011	No outcomes
Gorska-Ciebiada M	2015	No vaccination rate
Hun Kim	2019	No separate data for people with Diabetes

Ishigami	2019	No vaccination rate
Jackson	2003	No separate data for people with Diabetes
Jackson	2005	No separate data for people with Diabetes
Jackson	2009	No outcomes
Jacupus	2011	No vaccination rate
Kawakami	2010	No separate data for people with Diabetes
Koivula	1997	No separate data for people with Diabetes
Kojimahara	2008	No outcomes
Kopp	2021	No outcomes
Lai	2008	No outcomes
Lamontagne	2008	No vaccination rate
Lawerence	2020	No separate data for people with Diabetes
Lehmann	2000	No separate data for people with Diabetes
Levy	2019	No separate data for people with Diabetes
Lindeburg	2001	No separate data for people with Diabetes
Lopez-de-Andres	2020	No outcomes
Lopez-de-Andres	2021	No outcomes
Ma	2021	No separate data for people with Diabetes
Manzur	2011	No separate data for people with Diabetes
Maruyama	2010	No separate data for people with Diabetes
McLaughlin JM	2018	No separate data for people with Diabetes
Morton	2017	No vaccination rate
Mykietiuk	2006	No separate data for people with Diabetes
Norton	2019	No outcomes
Ochoa-Gondar O	2008	No separate data for people with Diabetes
Ochoa-Gondar O	2014	No separate data for people with Diabetes

Ortqvist	1998	No separate data for people with Diabetes
Papadatou	2016	No vaccination rate
Pebody	2008	No outcomes
Pekka O. Honkanen	1999	No separate data for people with Diabetes
Perniciaro	2021	No separate data for people with Diabetes
Prato	2018	No separate data for people with Diabetes
Principi N	2016	No outcomes
Ren	2018	No vaccination rate
Richard C	2016	No outcomes
Rivero-Calle	2021	No outcomes
Rodriguez	2014	No separate data for people with Diabetes
Rudnick W	2013	No separate data for people with Diabetes
Ryan	2001	No separate data for people with Diabetes
Salarbaks	2020	No outcomes
Seminog	2013	No outcomes
Shimbashi R	2020	No separate data for people with Diabetes
Stack	1999	No separate data for people with Diabetes
Steentoft	2007	No separate data for people with Diabetes
Su WJ	2021	No separate data for people with Diabetes
Sumitani	2008	No separate data for people with Diabetes
Suzuki	2017	No separate data for people with Diabetes
Vila Corcoles	2020	No outcomes
Vila-Corcoles	2004	No separate data for people with Diabetes
Vila-Corcoles	2006	No outcomes
Vila-Corocoles	2009	No separate data for people with Diabetes
Vila-Corocoles	2010	No separate data for people with Diabetes
Willis	2019	No outcomes

Yeon Heo	2022	No separate data for people with Diabetes
Zens	2022	No outcomes
Zhang	2020	No outcomes

Table 4S: List of excluded studies from the meta-analysis assessing the prognosis of pneumococcal disease in people with or without diabetes mellitus

Study	Adjustments	NOS Selection				NOS Comp	NOS Exp		
		1	2	3	4		1	2	3
Austrian1964	-	0	1	0	1	1	1	0	0
Aydin 2023	-	1	1	1	1	1	1	1	1
Baxter 2016	Age, race, vaccination, sex, year comorbidities	1	1	1	1	2	1	1	1
Cardinal-Fernández 2013	-	1	1	1	1	0	1	0	0
Di Yacovo 2013	-	1	1	1	1	0	1	1	1
Fedson 1983	-	0	1	1	1	1	1	0	0
Feikin 2000	-	1	1	1	1	0	1	1	1
Flory 2009	Age, sex, year, comorbidities	1	1	1	1	2	1	1	1
Fukuda 2022	-	1	1	1	1	0	1	1	1
Garrouste-Orgeas 2018	SOFA, lactate	1	1	1	1	2	1	1	1
Gil-Prieto 2016	-	1	1	0	1	0	1	1	0
Inghammar 2013	Comorbidity, education, socio-economic data	1	1	1	1	2	1	1	1
Ishiguro 2016	Age, PS, comorbidities	1	1	1	1	2	1	1	1
Jacups 2011	Age, sex, Indigenous, DM, smoking, alcohol	1	1	1	1	1	1	1	1
Klemets 2010	-	1	0	1	1	1	1	1	1
Kornum 2007	sex, age, comorbidity, alcohol, antibiotics,	1	1	1	1	1	1	1	1
Kwak 2015	Age and sex	0	1	1	1	1	1	0	1
Kyaw 2005	Age, race, comorbidities	1	1	0	1	1	0	1	1
Leelarasamee 1999	-	1	1	1	1	0	1	1	1
Lin 2019	Demographic characteristics	1	1	1	1	1	1	1	1
Lipsky 1986	-	1	0	1	1	1	1	1	1
Lòpez-De-Andrés 2017	-	1	1	1	1	1	1	1	1

Marrie 2011	Age and sex	0	1	1	1	0	1	0	1
Morrill 2014	-	1	1	1	1	1	0	0	0
Mufson 1974	-	1	1	1	1	0	1	1	1
Rueda 2009	Age, diabetes status, SMART-COP score	1	0	0	1	2	1	1	1
Seminog 2013	Age, sex, period, region, deprivation score	1	0	1	1	0	1	1	1
Shea 2014	-	1	1	1	1	1	1	1	1
Thomsen 2004		1	1	1	1	1	1	1	1
van Hoeak 2012		1	1	1	1	1	1	1	1
Vila-Corcoles 2015	Age, sex, comorbidities	1	1	1	1	1	1	1	1
Wagenvoort 2016	-	1	1	1	1	2	1	1	1
Watt 2007		1	0	1	1	1	1	1	1
Watanakunakorn 1993	Age, sex, comorbidities, smoking, alcohol fragility	1	1	1	1	0	1	1	1
Watanakunakorn 1997	Age, comorbidities	1	1	1	1	0	1	1	1
Willis 2019	-	1	1	1	1	0	1	1	1

Table 5S – Risk of bias of the included studies assessing the prognosis of pneumococcal disease in people with or without diabetes mellitus. *NOS* = Newcastle-Ottawa Scale, *Comp* = comparability of groups, *Exp* = exposition.

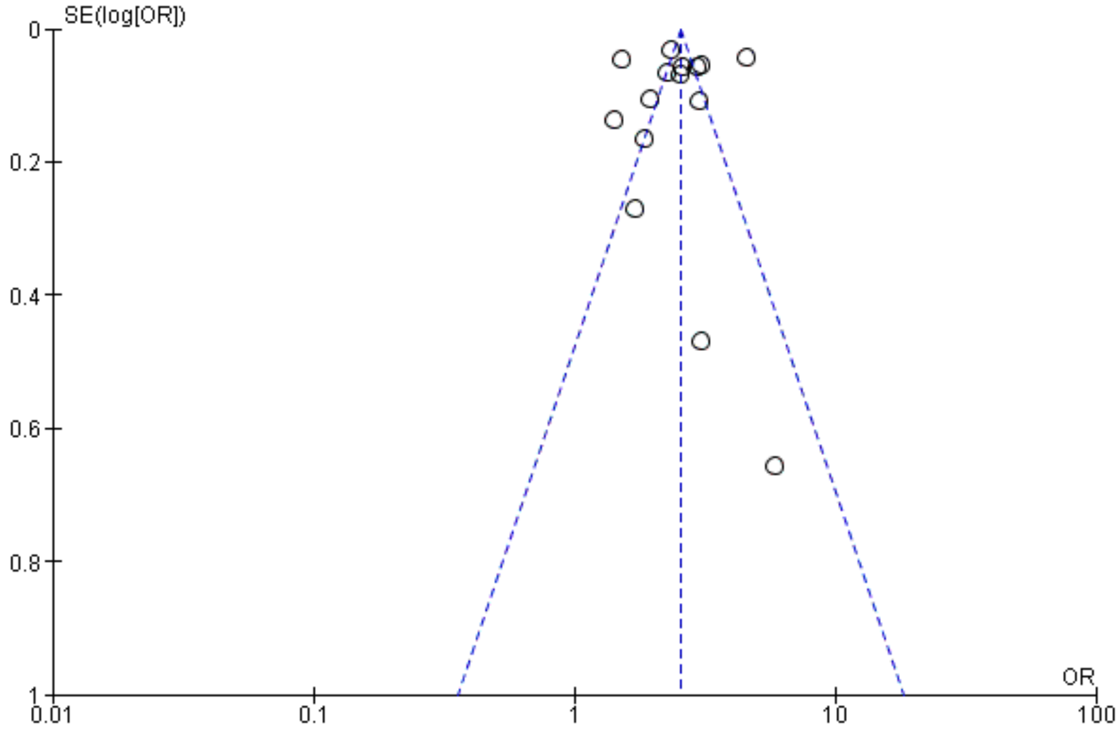

Figure 2S – Funnel plot for the incidence of invasive pneumococcal disease between people with or without diabetes mellitus

Study left out	OR for the remainder studies
Aydin 2023	2.54 [2.46, 2.63]
Baxter 2016	2.51 [2.42, 2.60]
Inghammar 2013	2.56 [2.48, 2.65]
Klemets 2010	2.55 [2.47, 2.64]
Kwak 2015	2.57 [2.48, 2.66]
Kyaw 2005	2.54 [2.46, 2.63]
Lin 2019	2.56 [2.48, 2.65]
Morril 2014	2.76 [2.66, 2.86]
Shea 2014	2.53 [2.44, 2.62]
Van Hoek 2012	2.29 [2.19, 2.39]
Wagenwoort 2016	2.50 [2.41, 2.59]
Watt 2007	2.55 [2.47, 2.64]

Table 6S –Leave-one out analysis the included studies assessing risk for invasive pneumococcal disease

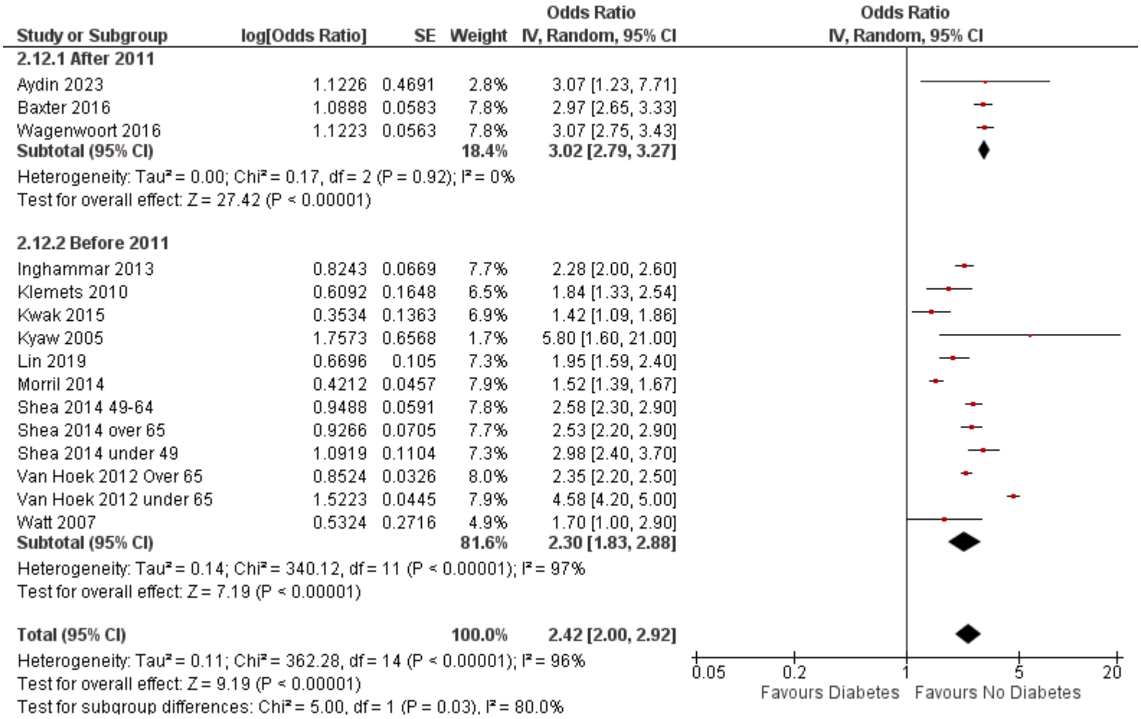

Figure 3S –Subgroup Analysis for invasive pneumococcal disease (IPD) in studies performed before or after 2011, *Unadjusted OR* (Forest plot, IV = inverse variance, CI = confidence interval)

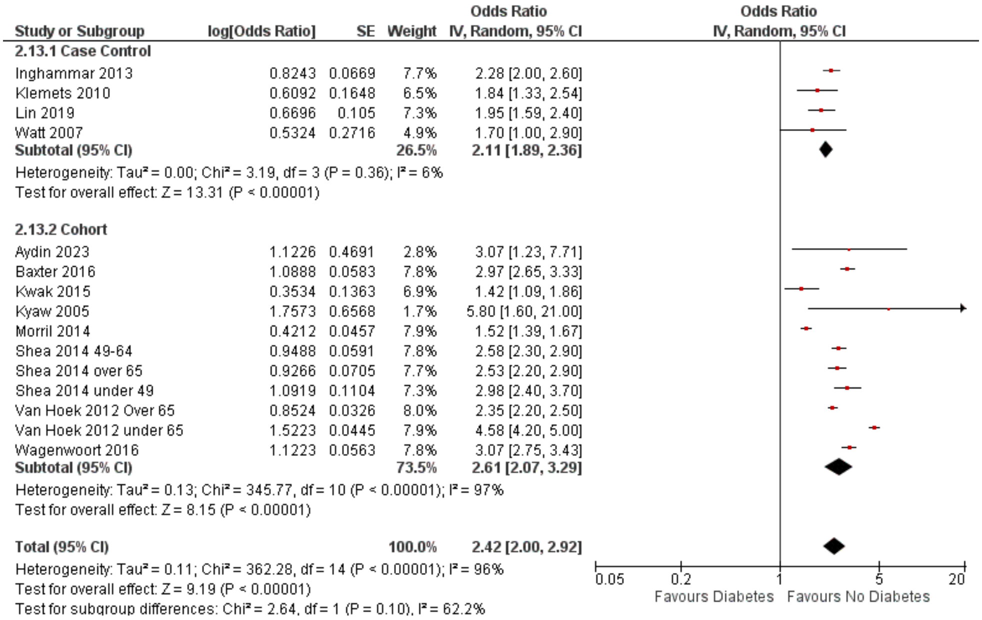

Figure 4S –Subgroup Analysis for invasive pneumococcal disease (IPD) for case-control or Cohort studies, *Unadjusted OR* (Forest plot, IV = inverse variance, CI = confidence interval)

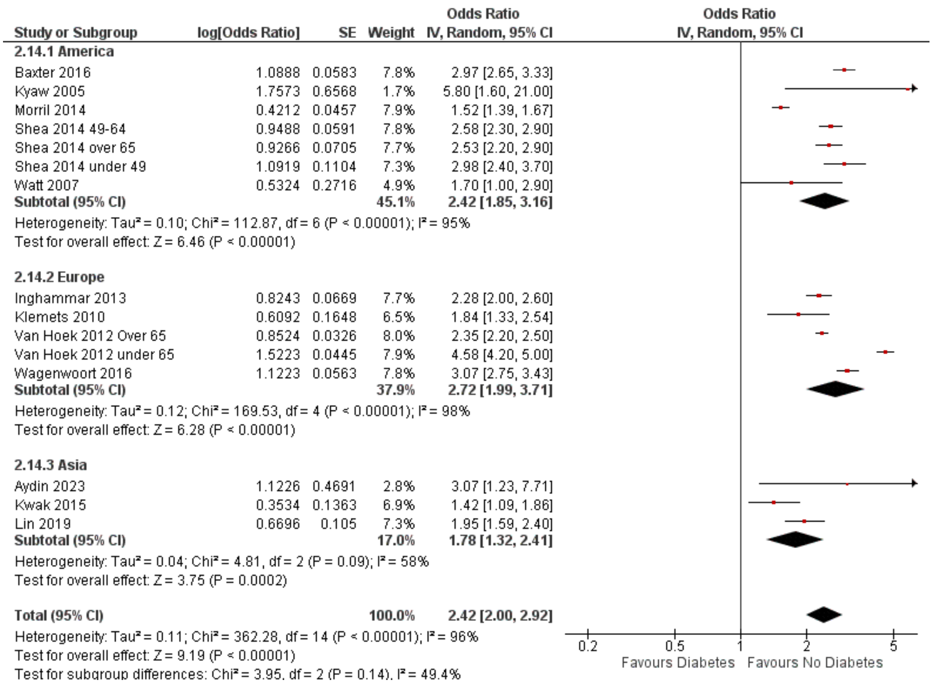

Figure 5S –Subgroup Analysis for invasive pneumococcal disease (IPD) for studies performed in different countries.

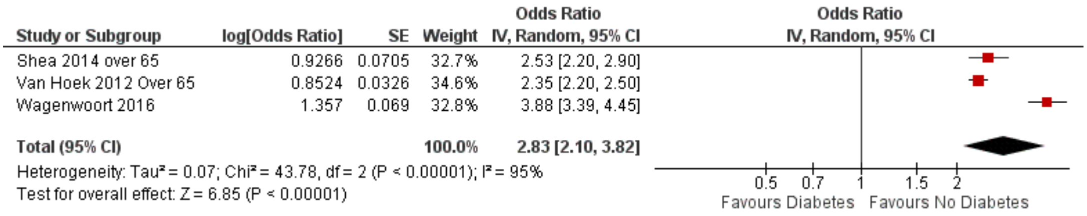

Figure 6S – Difference in risk for invasive pneumococcal disease (IPD) between patients aged over 65 with or wthout diabetes, *Unadjusted OR* (Forest plot, IV = inverse variance, CI = confidence interval)

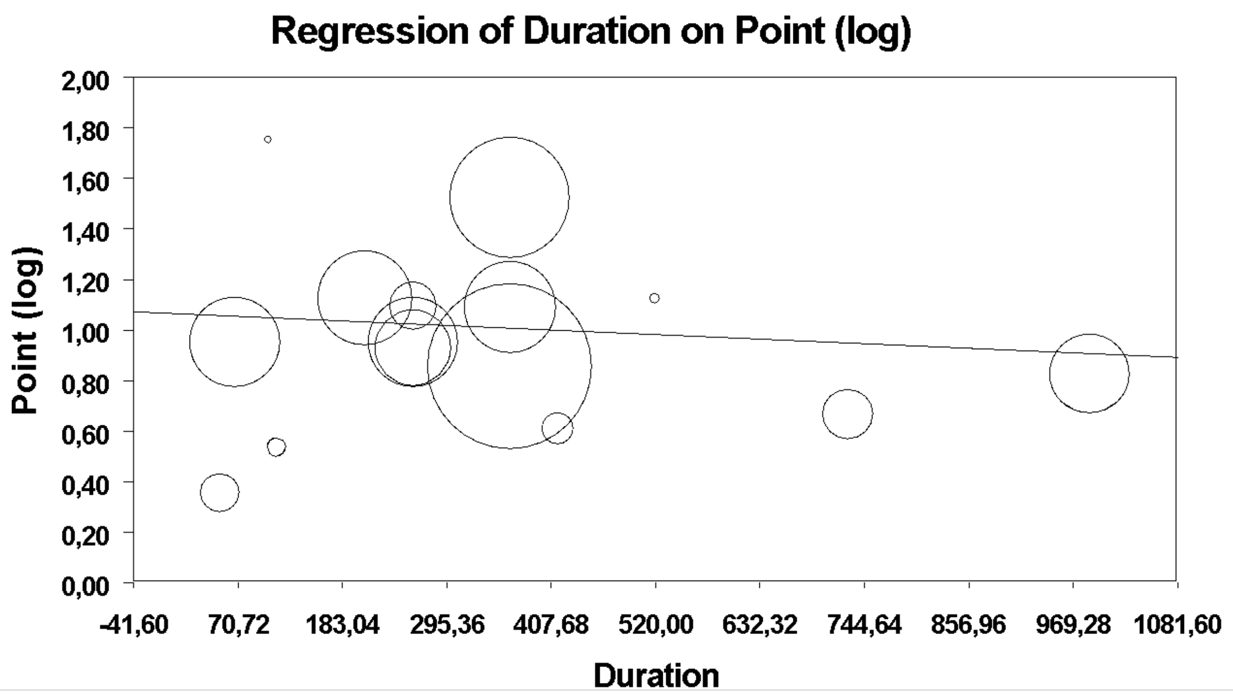

Figure 7S – Metaregression: Correlation between the effect of diabetes on the risk for invasive pneumococcal disease and the duration of the study (expressed as log OR) expressed in weeks

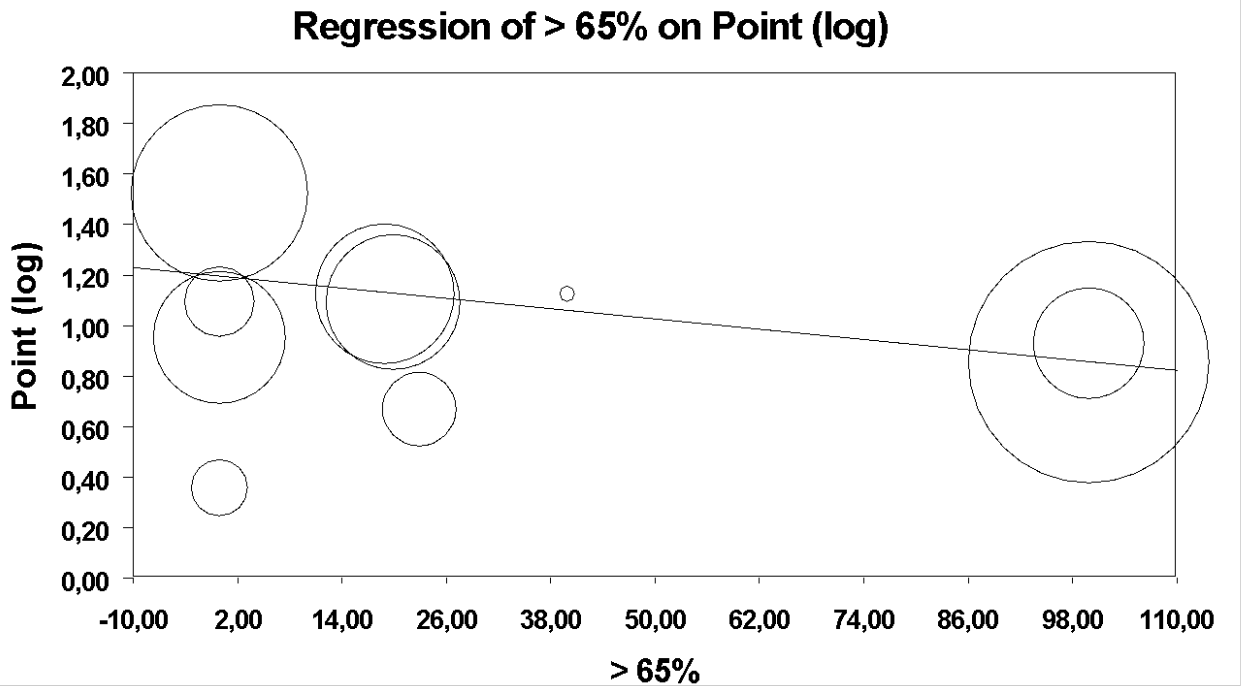

Figure 8S – Metaregression: Correlation between the effect of diabetes on the risk for invasive pneumococcal disease (expressed as log OR) and the proportion of individuals older than 65 years (expressed as %).

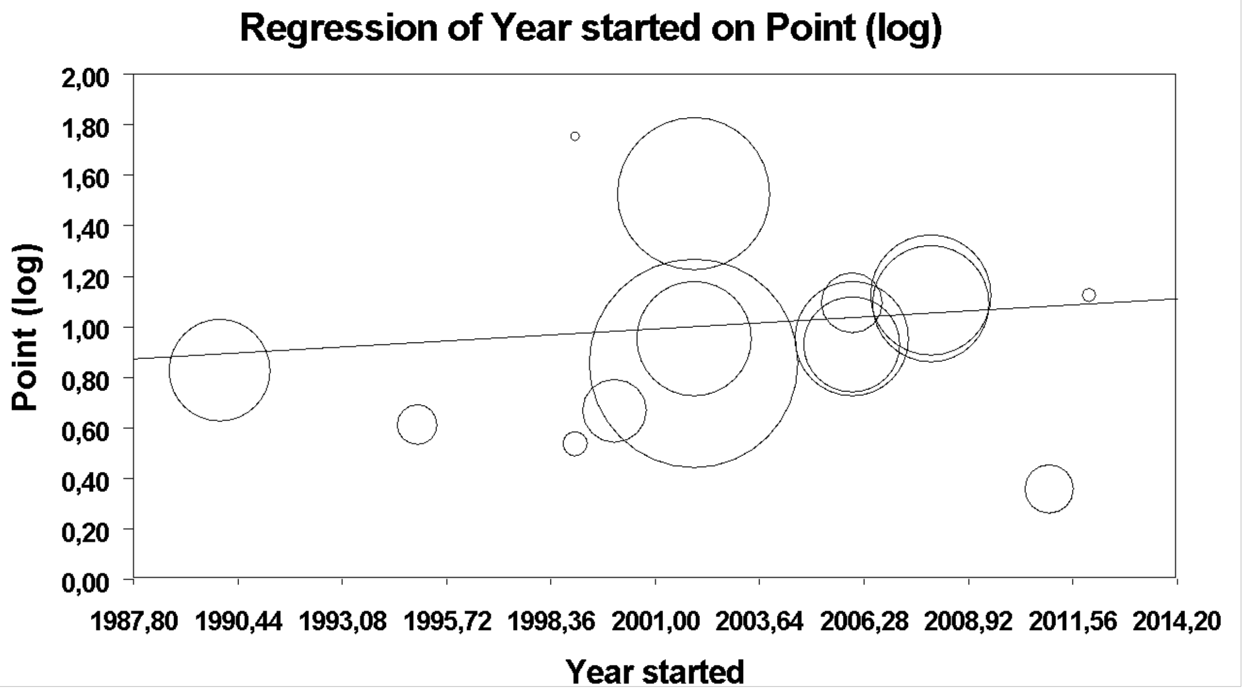

Figure 9S – Metaregression: Correlation between the effect of diabetes on the risk for invasive pneumococcal disease (expressed as log OR) and the year in which the study started.

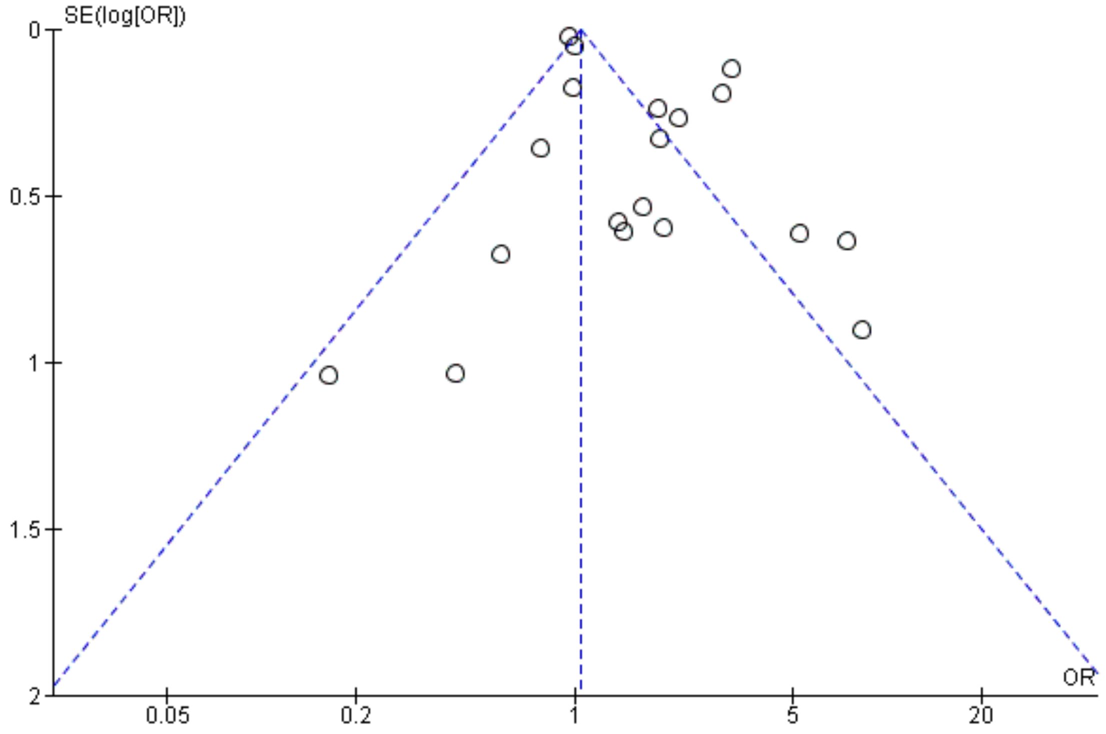

Figure 10S – Funnel plot for the effect on diabetes on case-fatality ratio (CFR) for pneumococcal disease

**Study left out**	**Unadj OR for the remainder studies**	**Adj OR for the remainder studies**
Austrian 1964	1.61 [1.24, 2.08]	
Aydin 2023	1.55 [1.20, 2.00]	
Cardinàl-Fernandez 2013	1.62 [1.25, 2.09]	
Fedson 1983	1.62 [1.25, 2.09]	
Feikin 2000	1.52 [1.18, 1.95]	
Garrouste-Orgeas 2018	1.57 [1.21, 2.04]	0.80 [0.42, 1.50]
Ishiguro 2015	1.64 [1.27, 2.11]	
Jacups 2011	1.61 [1.25, 2.07]	
Leelarasamee 1999	1.54 [1.20, 1.98]	
Lopez- De Andres 2017	1.70 [1.19, 2.42]	
Marrie 2011	1.59 [1.22, 2.07]	0.79 [0.43, 1.46]
Mufson 1974	1.60 [1.24, 2.07]	
Rueda 2009		1.13 [0.69, 1.87]
Thomsen 2004	1.68 [1.30, 2.18]	1.06 [0.58, 1.95]
Van Hoek 2012 Over 65	1.69 [1.17, 2.44]	

Van Hoek 2012 under 65	1.44 [1.17, 1.78]	
Wagenwoort 2016	1.69 [1.29, 2.21]	0.81 [0.60, 1.10]
Watanakunakorn 1993	1.59 [1.23, 2.07]	
Watanakunakorn 1997	1.66 [1.28, 2.14]	
Willis 2019	1.56 [1.22, 2.01]	

Table 7S –Leave-one out analysis the included studies assessing risk for Case-fatality Rate

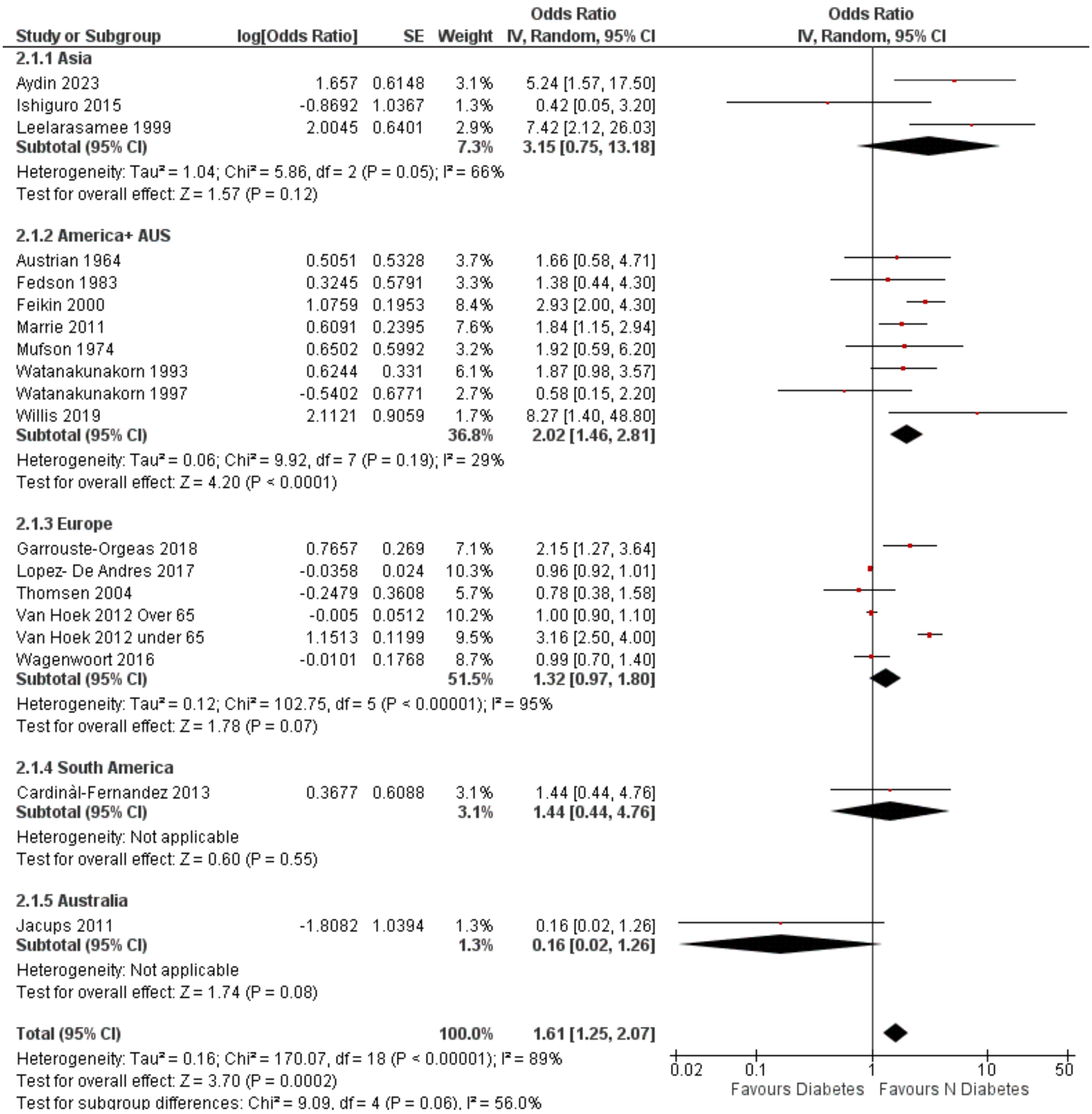

Figure 11S – Subgroup Analysis for CFR in studies performed in different countries (Forest plot, IV = inverse variance, CI = confidence interval)

Study or Subgroup	log[Odds Ratio]	SE	Weight	Odds Ratio IV, Random, 95% CI
**2.10.1 after 2004**				
Aydin 2023	1.657	0.6148	3.1%	5.24 [1.57, 17.50]
Cardinàl-Fernandez 2013	0.3677	0.6088	3.1%	1.44 [0.44, 4.76]
Ishiguro 2015	-0.8692	1.0367	1.3%	0.42 [0.05, 3.20]
Lopez- De Andres 2017	-0.0358	0.024	10.3%	0.96 [0.92, 1.01]
Van Hoek 2012 Over 65	-0.005	0.0512	10.2%	1.00 [0.90, 1.10]
Van Hoek 2012 under 65	1.1513	0.1199	9.5%	3.16 [2.50, 4.00]
Wagenwoort 2016	-0.0101	0.1768	8.7%	0.99 [0.70, 1.40]
Willis 2019	2.1121	0.9059	1.7%	8.27 [1.40, 48.80]
**Subtotal (95% CI)**			**47.9%**	**1.46 [1.05, 2.04]**
Heterogeneity: Tau² = 0.13; Chi² = 107.97, df = 7 (P < 0.00001); I² = 94%				
Test for overall effect: Z = 2.26 (P = 0.02)				
**2.10.2 Before 1980**				
Austrian 1964	0.5051	0.5328	3.7%	1.66 [0.58, 4.71]
Fedson 1983	0.3245	0.5791	3.3%	1.38 [0.44, 4.30]
Mufson 1974	0.6502	0.5992	3.2%	1.92 [0.59, 6.20]
**Subtotal (95% CI)**			**10.3%**	**1.63 [0.86, 3.11]**
Heterogeneity: Tau² = 0.00; Chi² = 0.15, df = 2 (P = 0.93); I² = 0%				
Test for overall effect: Z = 1.50 (P = 0.13)				
**2.10.3 1980-2005**				
Feikin 2000	1.0759	0.1953	8.4%	2.93 [2.00, 4.30]
Garrouste-Orgeas 2018	0.7657	0.269	7.1%	2.15 [1.27, 3.64]
Jacups 2011	-1.8082	1.0394	1.3%	0.16 [0.02, 1.26]
Leelarasamee 1999	2.0045	0.6401	2.9%	7.42 [2.12, 26.03]
Marrie 2011	0.6091	0.2395	7.6%	1.84 [1.15, 2.94]
Thomsen 2004	-0.2479	0.3608	5.7%	0.78 [0.38, 1.58]
Watanakunakorn 1993	0.6244	0.331	6.1%	1.87 [0.98, 3.57]
Watanakunakorn 1997	-0.5402	0.6771	2.7%	0.58 [0.15, 2.20]
**Subtotal (95% CI)**			**41.9%**	**1.70 [1.07, 2.70]**
Heterogeneity: Tau² = 0.27; Chi² = 24.19, df = 7 (P = 0.001); I² = 71%				
Test for overall effect: Z = 2.23 (P = 0.03)				
**Total (95% CI)**			**100.0%**	**1.61 [1.25, 2.07]**
Heterogeneity: Tau² = 0.16; Chi² = 170.07, df = 18 (P < 0.00001); I² = 89%				
Test for overall effect: Z = 3.70 (P = 0.0002)				
Test for subgroup differences: Chi² = 0.29, df = 2 (P = 0.87), I² = 0%				

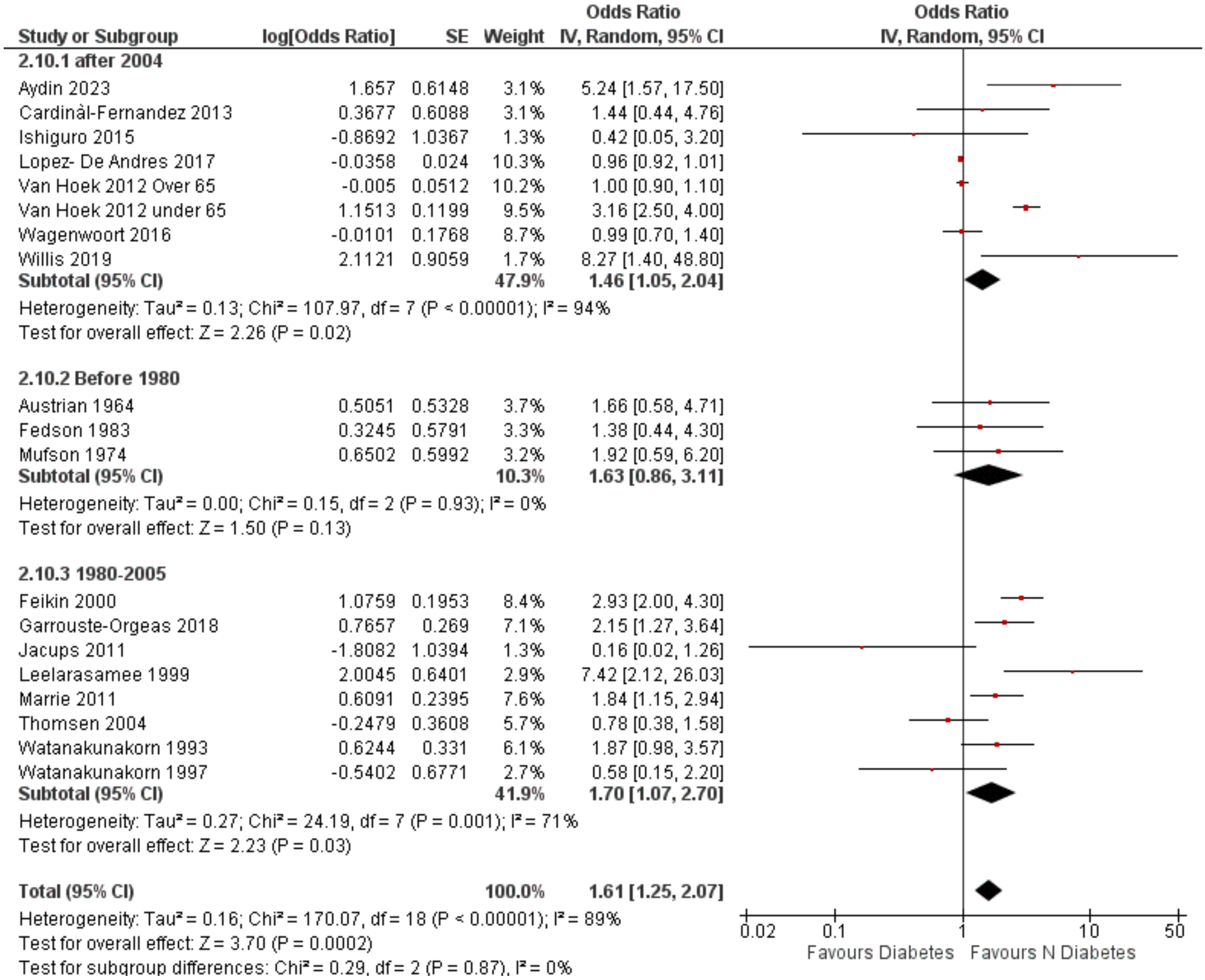

Figure 12S – Subgroup Analysis for CFR in studies performed before or after , *OR Unadjusted* Unadjusted OR (Forest plot, IV = inverse variance, CI = confidence interval)

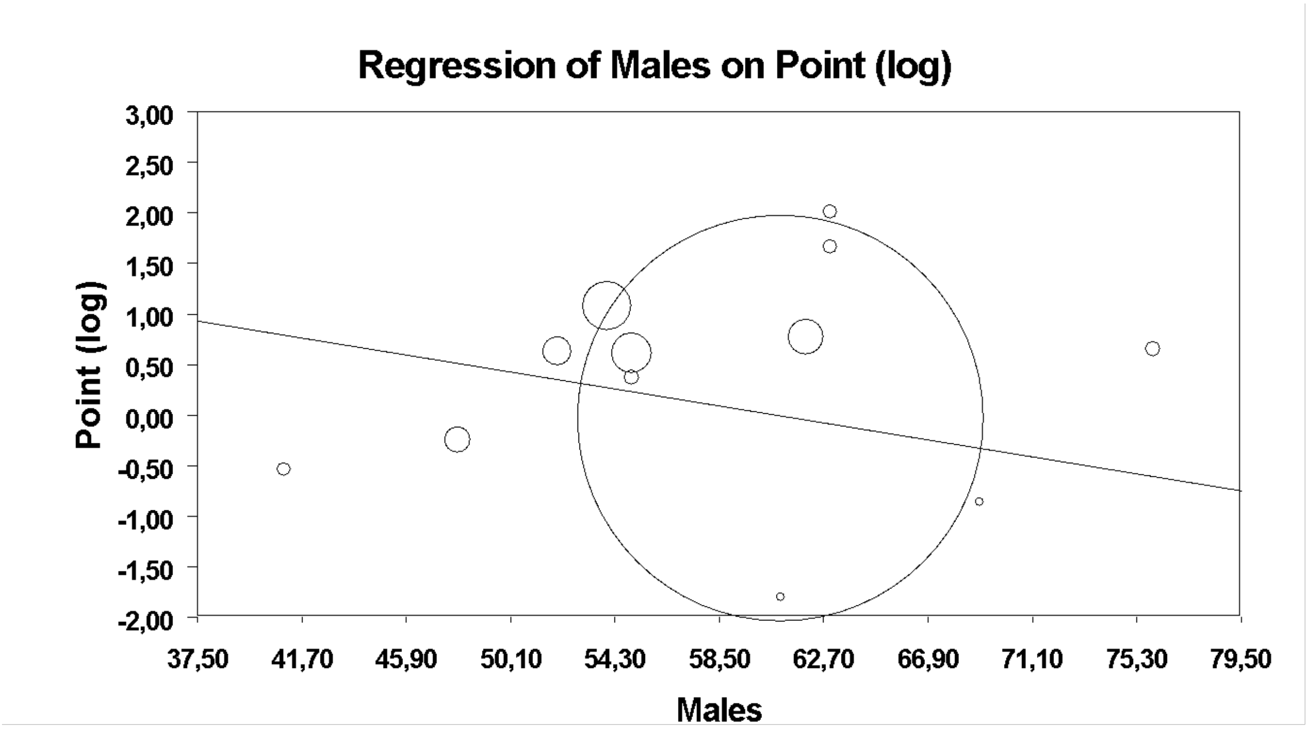

Figure 13S Metaregression: Correlation between the effect of diabetes on the risk for invasive pneumococcal disease (expressed as log OR) and the proportion of male (espressed as %) of individuals enrolled in each study.

Outcome or Subgroup	N Studies	Statistical Method	Effect Estimate
ICU admission	2	Unadj Odds Ratio (IV, Random, 95% CI)	2.09 [1.20, 3.66]
pneumococcal disease	3	Unadj Odds Ratio (IV, Random, 95% CI)	2.09 [1.20, 3.66]
pneumococcal disease (over 65 patients)	2	Unadj Odds Ratio (IV, Random, 95% CI)	2.09 [1.20, 3.66]
bacteraemia, septicaemia, meningitidis	2	Unadj Odds Ratio (IV, Random, 95% CI)	1.16 [0.56, 2.41]
bacteraemia, septicaemia, meningitidis	3	Adj Odds Ratio (IV, Random, 95% CI)	1.48 [1.08, 2.04]

Table 8S –Odd ratios for outcomes with a lower number of studies retrieved

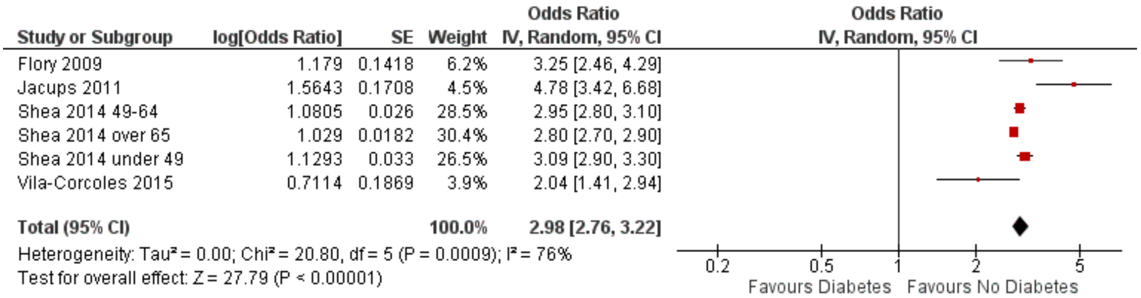

A

B

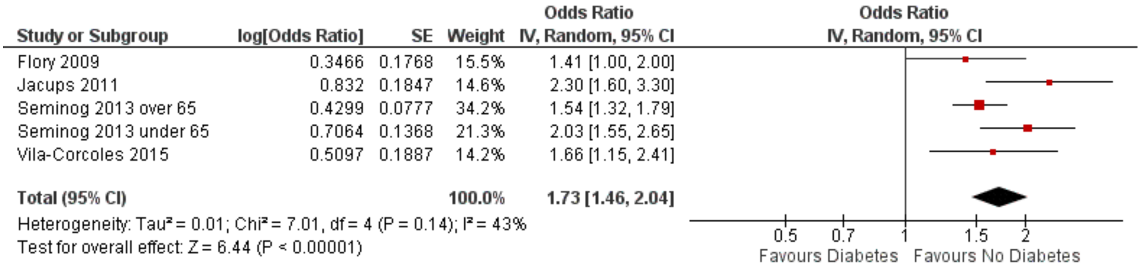

Figure 14S – Difference in risk for pneumococcal pneumonia between patients with or without diabetes, (Forest plot, IV = inverse variance, CI = confidence interval) **A** *Unadjusted OR,* **B** *Adjusted OR*

**Study**	**Year**	**Reason for exclusion**
Alfageme	2006	No separate data for people with Diabetes
Almusalam	2019	No outcomes
Andrews	2012	No separate data for people with Diabetes
Ansladi	2005	No separate data for people with Diabetes
Appel	2006	No outcomes
Aubert	2010	No outcomes
Bardenheier	2022	No outcomes
Bliss	2008	No separate data for people with Diabetes
Boey	2021	No outcomes
Chandler	2022	No separate data for people with Diabetes
Chen YH	2006	No outcomes
Chevalier-Cottin	2020	No outcomes
Christenson	2001	No separate data for people with Diabetes
Christenson	2004	No separate data for people with Diabetes
Christenson	2008	No separate data for people with Diabetes
Demirci	2021	No outcomes
Djennad A	2019	No separate data for people with Diabetes
Dominguez	2005	No separate data for people with Diabetes
Dorner	2011	No outcomes
Dower	2011	No outcomes
Furumoto	2008	No separate data for people with Diabetes
Gessner	2019	Double
Gil-Prieto R	2016	No vaccination rate
Gilbertson	2011	No outcomes
Gorska-Ciebiada M	2015	No vaccination rate
Hun Kim	2019	No separate data for people with Diabetes
Ishigami	2019	No vaccination rate
Jackson	2003	No separate data for people with Diabetes
Jackson	2005	No separate data for people with Diabetes
Jackson	2009	No outcomes
Jacupus	2011	No vaccination rate
Kawakami	2010	No separate data for people with Diabetes
Koivula	1997	No separate data for people with Diabetes
Kojimahara	2008	No outcomes
Kopp	2021	No outcomes
Lai	2008	No outcomes
Lamontagne	2008	No vaccination rate
Lawerence	2020	No separate data for people with Diabetes
Lehmann	2000	No separate data for people with Diabetes
Levy	2019	No separate data for people with Diabetes
Lindeburg	2001	No separate data for people with Diabetes
Lopez-de-Andres	2020	No outcomes

Lopez-de-Andres	2021	No outcomes
Ma	2021	No separate data for people with Diabetes
Manzur	2011	No separate data for people with Diabetes
Maruyama	2010	No separate data for people with Diabetes
McLaughlin JM	2018	No separate data for people with Diabetes
Morton	2017	No vaccination rate
Mykietiuk	2006	No separate data for people with Diabetes
Norton	2019	No outcomes
Ochoa-Gondar O	2008	No separate data for people with Diabetes
Ochoa-Gondar O	2014	No separate data for people with Diabetes
Ortqvist	1998	No separate data for people with Diabetes
Papadatou	2016	No vaccination rate
Pebody	2008	No outcomes
Pekka O. Honkanen	1999	No separate data for people with Diabetes
Perniciaro	2021	No separate data for people with Diabetes
Prato	2018	No separate data for people with Diabetes
Principi N	2016	No outcomes
Ren	2018	No vaccination rate
Richard C	2016	No outcomes
Rivero-Calle	2021	No outcomes
Rodriguez	2014	No separate data for people with Diabetes
Rudnick W	2013	No separate data for people with Diabetes
Ryan	2001	No separate data for people with Diabetes
Salarbaks	2020	No outcomes
Seminog	2013	No outcomes
Shimbashi R	2020	No separate data for people with Diabetes
Stack	1999	No separate data for people with Diabetes
Steentoft	2007	No separate data for people with Diabetes
Su WJ	2021	No separate data for people with Diabetes
Sumitani	2008	No separate data for people with Diabetes
Suzuki	2017	No separate data for people with Diabetes
Vila Corcoles	2020	No outcomes
Vila-Corcoles	2004	No separate data for people with Diabetes
Vila-Corcoles	2006	No outcomes
Vila-Corocoles	2009	No separate data for people with Diabetes
Vila-Corocoles	2010	No separate data for people with Diabetes
Willis	2019	No outcomes
Yeon Heo	2022	No separate data for people with Diabetes
Zens	2022	No outcomes
Zhang	2020	No outcomes

Table 9S: List of articles excluded for the study on the effectiveness of pneumococcal vaccination in people with diabetes with the reason for exclusion.

Study	Adjustments	NOS Sel				NOS Comp	NOS Exp		
		1	2	3	4		1	2	3
Benin 2003	Age, sex, comorbidity	.	.	.	.	. .	.	.	.
Butler 1993	Age, sex, comorbidity, residence	.	.	.	.		.	.	

Davis 2017	Age, BMI, home health care, albuminuria, comorbidities	.	.	.	.	. .	.	-	-
Fisman 2006	Age, PORT score, hospitalization duration	.	.	.	.	.	.	.	-
Hsiao 2022	Age at vaccination, sex, smoking, vaccine, comorbidities	.	.	.	.	.	.	.	.
Kuo 2016	Age	.	.	.	.	. .	.	.	.
Mc Donald 2017	Age,sex, dm duration, MPR, comorbidities, vaccination, P4P,	.	.	.	.	. .	.	.	.
Skull 2006	Age, sex, socioeconomic, smoking, comorbidities, therapy, Hb1Ac	.	.	.	.	. .	.	.	.
Vila-Corocoles 2019	Residence, timing, propensity score	.	.	.	.	. .	.	.	.
Wagner 2003	Age, sex, vaccine, comorbidities, smoking, alcohol,	-	.	.	.	.	.	.	.

Table 10S: Risk of bias of observational studies investigating the efficacy of pneumococcal vaccine in people with diabetes (NOS= Newcastle-Ottawa Scale; Selection= selection of study groups; compar= comparability of groups; Exp= exposure

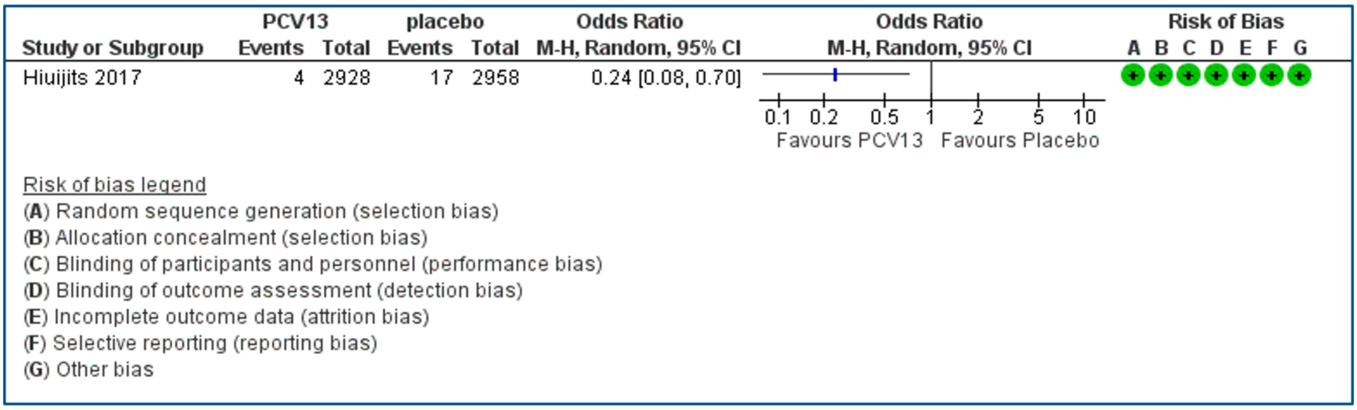

Figure 15S: Differences in vaccine-type pneumonia hospitalizations between vaccinated or unvaccinated patients with diabetes (forest plot; IV= Inverse Variance Random = random effects CI = Confidence interval).

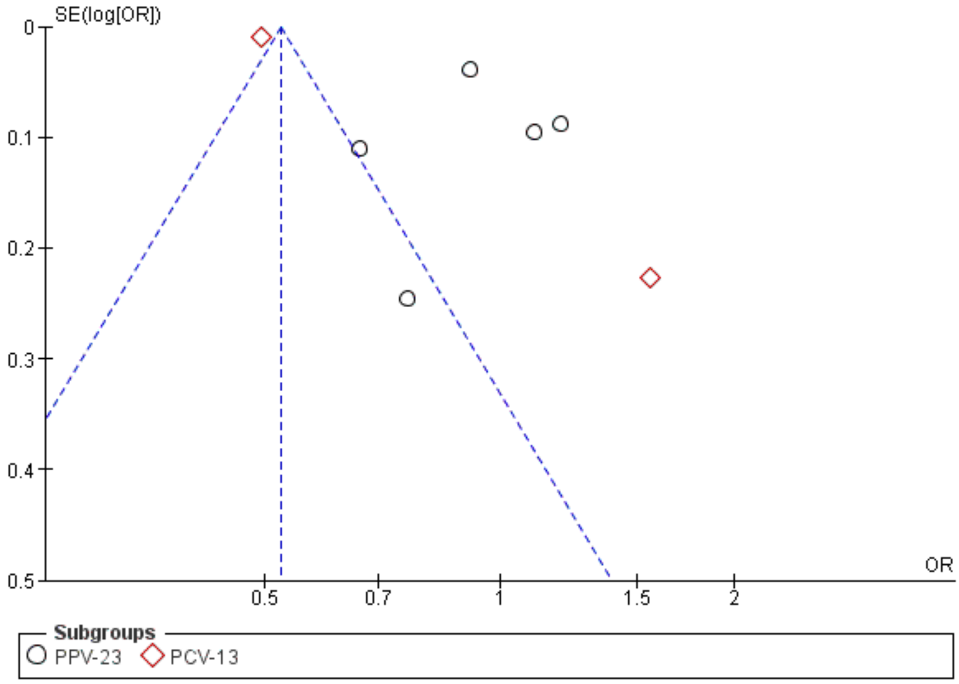

Figure 16S: funnel plot for pneumonia hospitalization rate in those with diabetes, with versus without pneumococcal vaccination

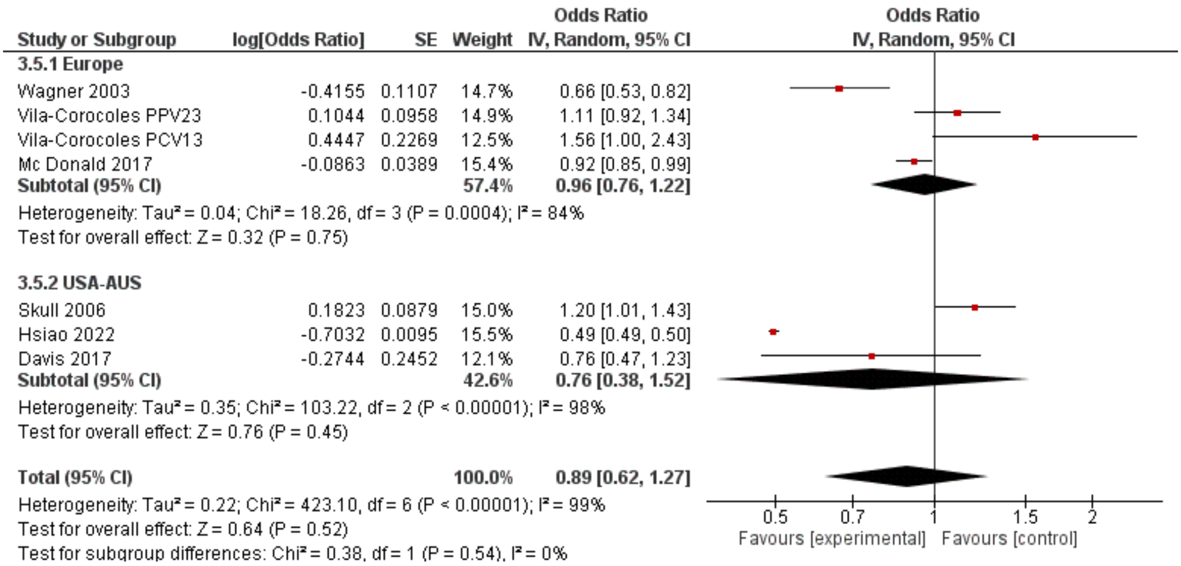

Figure 17S: Subgroup analysis for pneumonia hospitalizations in people with diabetes mellitus with or without vaccination, studies performed inside or outside Europe.

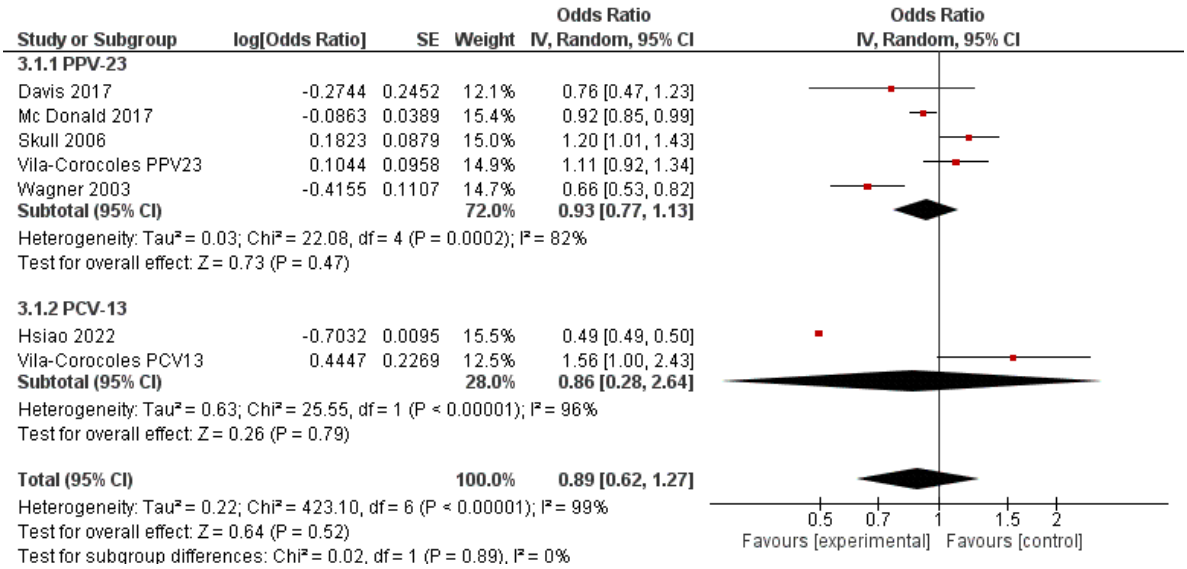

Figure 18S: Subgroup analysis for pneumonia hospitalizations in people with diabetes mellitus with or without vaccination, studies are grouped according to the type of anti-pneumococcal vaccination performed: PPV-23 polysaccharide vaccine and PCV13 conjugate vaccine.

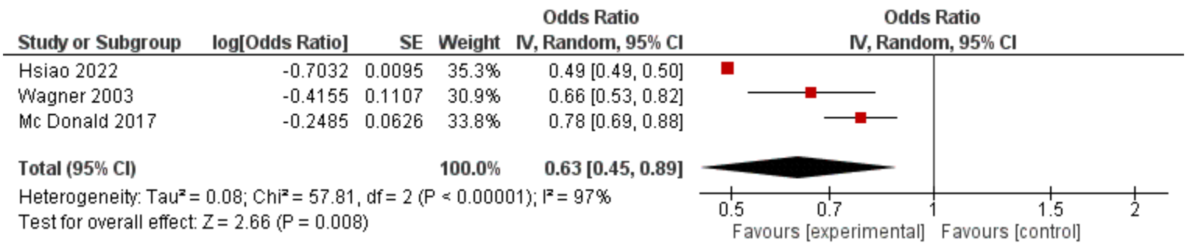

Figure 19S: Subgroup analysis for pneumonia hospitalizations including diabetic individuals who have been vaccinated for less than one year and diabetic individuals who have not been vaccinated.

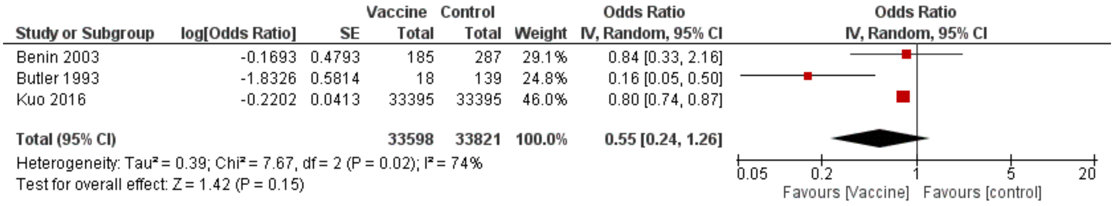

Figure 20S Differences in Invasive Pneumococcal Disease incidence between vaccinated or unvaccinated patients with diabetes (forest plot; IV= Inverse Variance Random = random effects CI = Confidence interval).

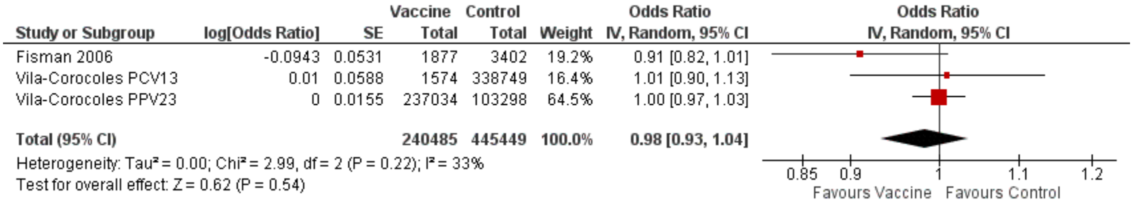

Figure 21S: Differences in overall mortality incidence between vaccinated or unvaccinated patients with diabetes (forest plot; IV= Inverse Variance Random = random effects CI = Confidence interval).
